# Gait changes over time in hospitalized older adults with advanced dementia: Predictors of mobility change

**DOI:** 10.1371/journal.pone.0259975

**Published:** 2021-11-17

**Authors:** Sina Mehdizadeh, Mohammadreza Faieghi, Andrea Sabo, Hoda Nabavi, Avril Mansfield, Alastair J. Flint, Babak Taati, Andrea Iaboni

**Affiliations:** 1 KITE- Toronto Rehabilitation Institute, University Health Network, Toronto, Ontario, Canada; 2 Evaluative Clinical Sciences, Hurvitz Brain Sciences Program, Sunnybrook Research Institute, Toronto, Ontario, Canada; 3 Department of Physical Therapy, University of Toronto, Toronto, Ontario, Canada; 4 Department of Psychiatry, University of Toronto, Toronto, Ontario, Canada; 5 Centre for Mental Health, University Health Network, Toronto, Ontario, Canada; 6 Institute of Biomaterials and Biomedical Engineering, University of Toronto, Toronto, Ontario, Canada; 7 Vector Institute for Artificial Intelligence, Toronto, Ontario, Canada; 8 Department of Computer Science, University of Toronto, Toronto, Ontario, Canada; Cardiff University, UNITED KINGDOM

## Abstract

People with dementia are at risk of mobility decline. In this study, we measured changes in quantitative gait measures over a maximum 10-week period during the course of a psychogeriatric admission in older adults with dementia, with the aims to describe mobility changes over the duration of the admission, and to determine which factors were associated with this change. Fifty-four individuals admitted to a specialized dementia inpatient unit participated in this study. A vision-based markerless motion capture system was used to record participants’ natural gait. Mixed effect models were developed with gait measures as the dependent variables and clinical and demographic variables as predictors. We found that gait stability, step time, and step length decreased, and step time variability and step length variability increased over 10 weeks. Gait stability of men decreased more than that of women, associated with an increased sacrum mediolateral range of motion over time. In addition, the sacrum mediolateral range of motion decreased in those with mild neuropsychiatric symptoms over 10 weeks, but increased in those with more severe neuropsychiatric symptoms. Our study provides evidence of worsening of gait mechanics and control over the course of a hospitalization in older adults with dementia. Quantitative gait monitoring in hospital environments may provide opportunities to intervene to prevent adverse events, decelerate mobility decline, and monitor rehabilitation outcomes.

## Introduction

Older adults with dementia have a higher frequency of hospitalization, and longer lengths of hospital stay compared to seniors without dementia [1]. People with behavioural and psychological symptoms of dementia (BPSD) are at particular risk of prolonged hospitalization and adverse effects related to the hospital stay [2]. Hospitalization can result in both positive and negative changes in mobility and functional status in older adults with dementia. That is, while factors such as polypharmacy, immobilization, isolation, and prolonged length of hospital stay can cause hospital-related mobility decline [3, 4], physical activity and rehabilitation programs may improve mobility [5]. Monitoring of mobility status in hospitalized older adults with dementia is important, because a decline in mobility not only has an impact on activities of daily living such as walking, toileting, and social engagement, but also increases the risk for falls, pressure ulcers, and incontinence, all of which can lead to long-term hospitalization or even death [6–9].

To address this issue, there is a need to monitor changes in mobility over time in hospitalized older adults with dementia. Several studies have measured mobility change in hospitalized older adults with dementia using activities of daily living assessment tools that incorporate an assessment of mobility, such as the Minimum Data Set, Care Dependency Scale, Barthel Index, or Katz Index [8]. These tools provide clinical observations about mobility status, but do not reveal the underlying biomechanical changes associated with mobility decline. For example, these methods will not reveal what aspects of the individual’s walking (e.g., walking speed or balance) are changing. These approaches may miss subtle changes occurring slowly over long periods of time and also rely on clinical judgement and expertise. Moreover, assessments can be infrequent [8, 10], and may thus be conducted too late to intervene if decline is rapid [11].

Recent advances in markerless motion capture technology allow for longitudinal, unobtrusive, and frequent daily-life assessment of gait over time. Using such a vision-based system, we recently demonstrated that impaired gait stability over a baseline period of two weeks is associated with future falls in a cohort of older adults with dementia with BPSD admitted to a specialized dementia unit [12, 13]. However, we have not previously examined the uses of this system for monitoring longitudinal changes and trajectory of participants’ daily-life gait, to establish whether it is possible to detect changes in mobility and identify clinical or demographic factors contributing to this change.

The aims of this study were, therefore, to i) describe changes in quantitative gait measures over 10 weeks in hospitalized older adults with advanced dementia and BPSD, and ii) determine which demographic and clinical factors were associated with gait changes over 10 weeks.

## Materials and methods

### Setting and participants

Data were collected during the participants’ inpatient stay in the Specialized Dementia Unit at Toronto Rehabilitation Institute-University Health Network. This is a psychogeriatric unit that admits people with dementia and severe BPSD from long-term care and acute care settings. The University Health Network (UHN) Research Ethics Board approved the study protocol. Substitute decision-makers provided written informed consent for all participants, and participants assented to all assessments. The inclusion criteria were diagnosis of dementia and ability to walk independently for 20 metres. The exclusion criterion was the use of any walking aids as our intention was to study participants’ ability in walking independently and its change over time.

### Gait data collection

We used a validated [14, 15] vision-based gait monitoring system to record natural bouts of walking of our participants. The system consisted of a Microsoft Kinect for Windows v2 mounted on the ceiling in a hallway. Previous studies have demonstrated good reliability of gait measures recorded using the Kinect v2 camera (intraclass correlation coefficient >0.73) [15–17]. An RFID system identified the participants, and custom-written software automatically turned on the camera when a participant wearing the RFID tag passed by the RFID antenna.

### Gait variables

The Kinect software development kit (SDK 2.0) was used to extract the 3D joint positions during walks. The following gait variables were calculated: spatiotemporal (gait speed, step time, length and width), variability (coefficient of variation (CV) of step time, length and width), range of motion of sacrum in mediolateral direction (sacrum ML ROM), and root mean square average of sacrum velocity in mediolateral direction (sacrum ML velocity RMS), and lateral mechanical stability (estimated margin of stability in the lateral direction, eMOS). eMOS measures the lateral distance between the velocity-corrected centre of mass (called XCOM) and the base of support (defined as the lateral position of the feet). The shorter the distance between the centre of mass and the base of support, the more likely the centre of mass will go beyond this base of support and lead to a fall. Thus, a lower value of eMOS is indicative of a lower stability and higher risk of fall. Thus, a lower value of the eMOS is indicative of a lower stability and higher risk of fall. It should be noted that sacrum ML ROM and sacrum ML velocity RMS were included in our analysis as the motion and velocity of the centre of mass (as approximated by sacrum) contribute to the assessment of eMOS and thus provides insight into the eMOS results. These gait variables were chosen to provide a comprehensive gait assessment and based on previous findings that impaired gait as estimated with these variables is associated with future falls in our cohort of participants [12, 18]. Gait variables were calculated for all recorded walks up to a maximum 10-week period for each participant. Matlab software (Mathwork Inc., Natick, USA) was used to calculate gait variables.

### Clinical assessments

At baseline, the following clinical assessments were completed: demographic data (sex and age), diagnosis of dementia based on geriatric psychiatric assessment, Neuropsychiatric Inventory (NPI) total score [19], history of falls in the previous 3 months, and functional mobility (Tinetti Performance Oriented Mobility Assessment balance component, POMA-balance [20]). These clinical variables were selected based on their known association with risk of falls in a previous study [12]. In addition, to investigate whether gait changes were associated with falls or medical events, falls during the 10-week period, and transfer to the emergency department or acute care hospitalization of the participants were included as time-varying covariates. Finally, the use of three separate groups of psychotropic medications (antipsychotics, benzodiazepines, and antidepressants) at baseline (the day of the first walk) was obtained from medical records and was included as a binary predictor in the model.

### Statistical analysis

To analyze the longitudinal change in gait over 10-weeks, mixed effect models (also known as linear growth models) were used. Mixed effect models allow modeling both fixed and random effects at two levels in repeated measures—level-1 (within-subject variability, usually over time) and level-2 (between-subject variability), and are particularly useful when the number of repeated measurements over time differs between participants [21, 22]. To analyze our data with the mixed effect models, three models were developed—unconditional means model (UMM), unconditional growth model (UGM), and finally conditional growth model (CGM, final model). In all three models, the gait variable was the dependent variable and the analysis was repeated for all gait variables described in section 2.3. As the first step, the UMM allowed us to understand the extent to which there was significant between-walk gait variability over time within and between individuals and thus providing the rationale for exploring predictors to help explain this variability. In the second step, the UGM was applied where the only predictor was time. This model established whether gait measures vary within individuals over time. The CGM was the final model in our mixed effect analysis where we added different clinical predictors introduced in section 2.4 to explain the variability in the gait measures. Our specific interest was if and how predictors affect change (slope) of gait measures over time (i.e., the interaction between each predictor and time).

## Results

### Participants

Fifty-four participants were included in the analysis. In total, participants had 4331 walks with the average (standard deviation) of 80.2 (68.4) walks per person and range of 8–306 walks. Data were collected over a mean of 6.0 weeks (standard deviation 2.4 weeks, range 1–10 weeks). Twenty-one participants (38.8%) had at least one fall over the 10 weeks, with 53 total falls and a range of 1–8 falls. In addition, 14 participants were transferred at least once to the emergency department. The reasons for hospital transfer were due to a new medical symptom or condition (infection, syncope, bowel obstruction, gastrointestinal bleed) or due to fall or fall-related injury. Participants’ demographic and clinical variables are summarized in Table 1. Accordingly, our participants had impaired balance as indicated by a mean POMA-balance score of 9.5 ± 3.1 and BPSD symptoms in the severe range with a mean NPI score of 52.1 ± 21.4. All participants had moderate to severe dementia as reflected by a mean Severe Impairment Battery Score [23] of 24.7 ± 16.3, and a mean Katz Index of Independence in Activities of Daily Living scale [24] of 2.2 ±1.4.

**Table 1:** Demographic and clinical variables (mean ± standard deviation) for study participants (n = 54).

Age (years)		76.3 ± 8.0
Men (N (%))		29 (53.8)
Number of walks		80.2 ± 68.4
Height (cm)		163.4 ± 14.4
Weight (kg)		66.9 ± 13.0
POMA-balance		9.5 ± 3.1
NPI		52.1 ± 21.4
Katz		2.2 ±1.4
SIBS		24.7 ± 16.3
Fall history (N (%))		18 (33.3)
Participants medication use at baseline (N (%))	Antipsychotics	44 (81.4)
	Benzodiazepines	18 (33.3)
	Antidepressants	31 (57.4)

POMA = Tinetti Performance Oriented Mobility Assessment; NPI = Neuropsychiatric Inventory score; Katz = Katz Index of Independence in Activities of Daily Living scale; SIBS = Severe Impairment Battery Score.

### Unconditional means model

The coefficient of regression for all gait variables was significantly different from zero (coefficients were eMOS (6.48 cm), step time (0.62 s), step length (30.68 cm), step time CV (22.27%), step length CV (26.83%), sacrum ML velocity RMS (17.90 cm/s), gait speed (49.61 cm/s), step width (16.77 cm), step width CV (32.90%,), and sacrum ML ROM (23.82 cm)). All p-values <0.001; S1 File). The UMM also demonstrated significant variability within and between participants in the study (S1 File) justifying the inclusion of predictors to explain these two types of variability.

### Unconditional growth model

**Fig 1:**
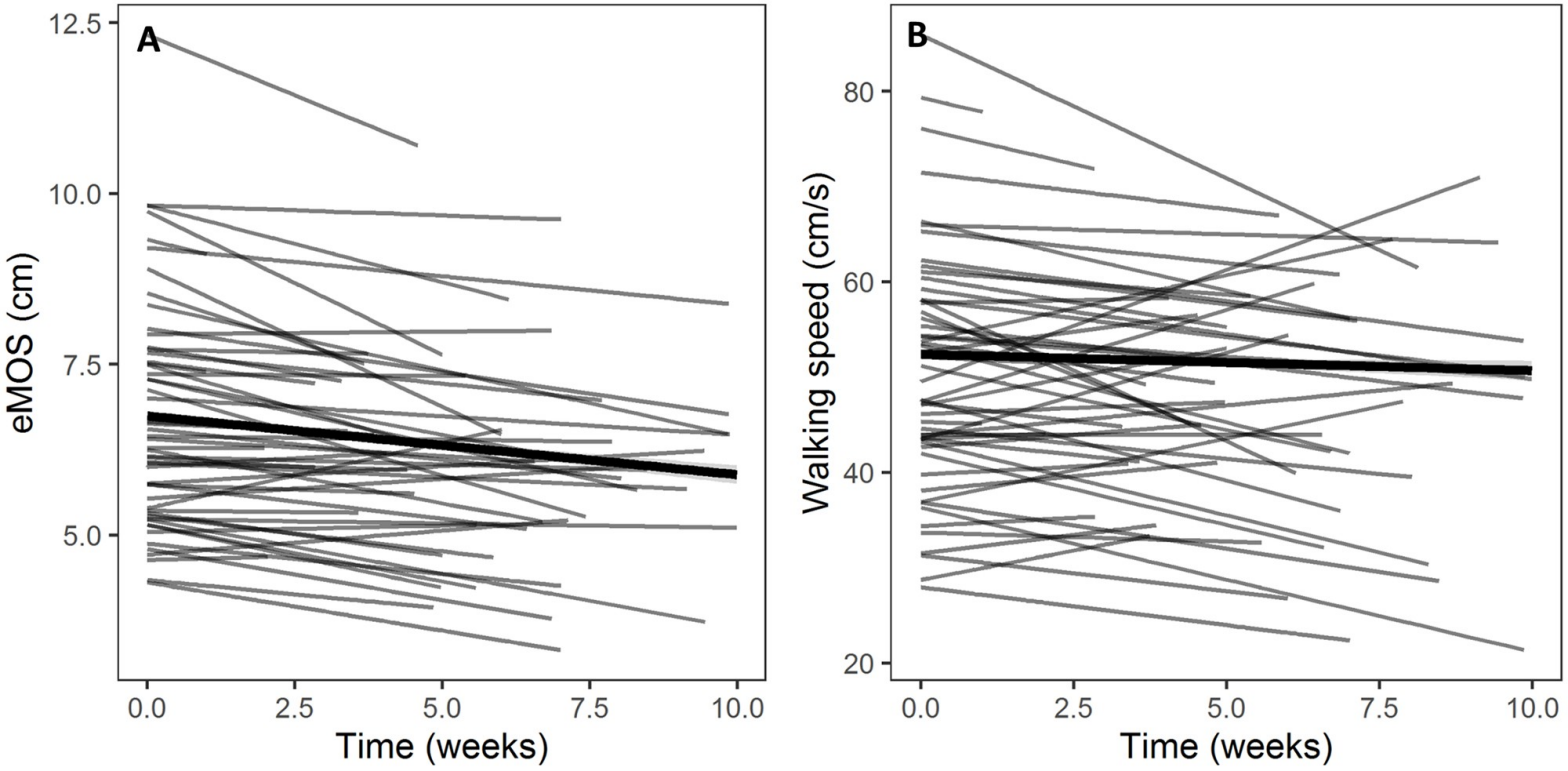
Spaghetti plot of A) estimated margin of stability (eMOS) and, B) walking speed developed by the unconditional growth model. The gray lines are for individuals and the thick black line is the average for all participants and all times. See section 3.3 and Table 2 for further explanations.

Of the ten gait measures examined, five changed over time unconditionally. Over the course of 10 weeks, three variables decreased unconditionally (Table 2 and S1 File): eMOS (coefficient = -0.09 cm/week, p = 0.003; Fig 1A), step time (coefficient = -0.004 s/week, p = 0.004), and step length (coefficient = -0.48 cm/week, p<0.001). Two variables increased unconditionally (Table 2 and S1 File): step time CV (coefficient = 1.18%/week, p<0.001) and step length CV (coefficient = 1.24%/week, p = 0.001). While sacrum ML velocity RMS (coefficient = 0.19 cm/s/week, p = 0.05), walking speed (coefficient = -0.43 cm/s/week, p = 0.07; Fig 1B), step width (coefficient = 0.06 cm/week, p = 0.34), step width CV (coefficient = 0.01%/week, p = 0.93) and sacrum ML ROM (coefficient = 0.40 cm/week, p = 0.13) did not significantly change over time, we included them in the subsequent CGM analyses as their variation over time might depend on clinical variables. It should be noted that in our preliminary analyses, we included age and sex as the predictors in the UGM. However, since there was no effect of these predictors or their interaction on the slope of the gait change, we excluded them from the UGM to keep the analyses simple and interpretable.

**Table 2:** The results of slope of change of gait variables (per week) using the unconditional growth model.

Gait variable	Coefficient	SD	CI	p-value
eMOS (cm/week)	-0.09	0.03	-0.16 –-0.03	**0.003**
Step time (s/week)	-0.004	0.001	-0.007 –-0.001	**0.004**
Step length (cm/week)	-0.48	0.12	-0.73 –-0.22	**<0.001**
Step time CV (%/week)	1.18	0.33	0.52–1.85	**<0.001**
Step length CV (%/week)	1.24	0.36	0.51–1.96	**0.001**
Sacrum ML velocity RMS (cm/s)	0.19	0.10	-0.002–0.39	0.05
Gait speed (cm/s/week)	-0.42	0.24	-0.90–0.04	0.07
Step width (cm/week)	0.05	0.06	-0.06–0.18	0.34
Step width CV (%/week)	0.01	0.20	-0.38–0.42	0.93
Sacrum ML ROM (cm/week)	0.40	0.27	-0.12–0.93	0.13

eMOS = estimated margin of stability; CV = coefficient of variation; Sacrum RMS ML velocity = root mean square average of sacrum velocity in mediolateral direction; Sacrum ML ROM = sacrum range of motion in mediolateral direction; SD = standard deviation; CI = 95% confidence interval; α = 0.05.

See the S1 File for complete results.

### Conditional growth model

**Fig 2:**
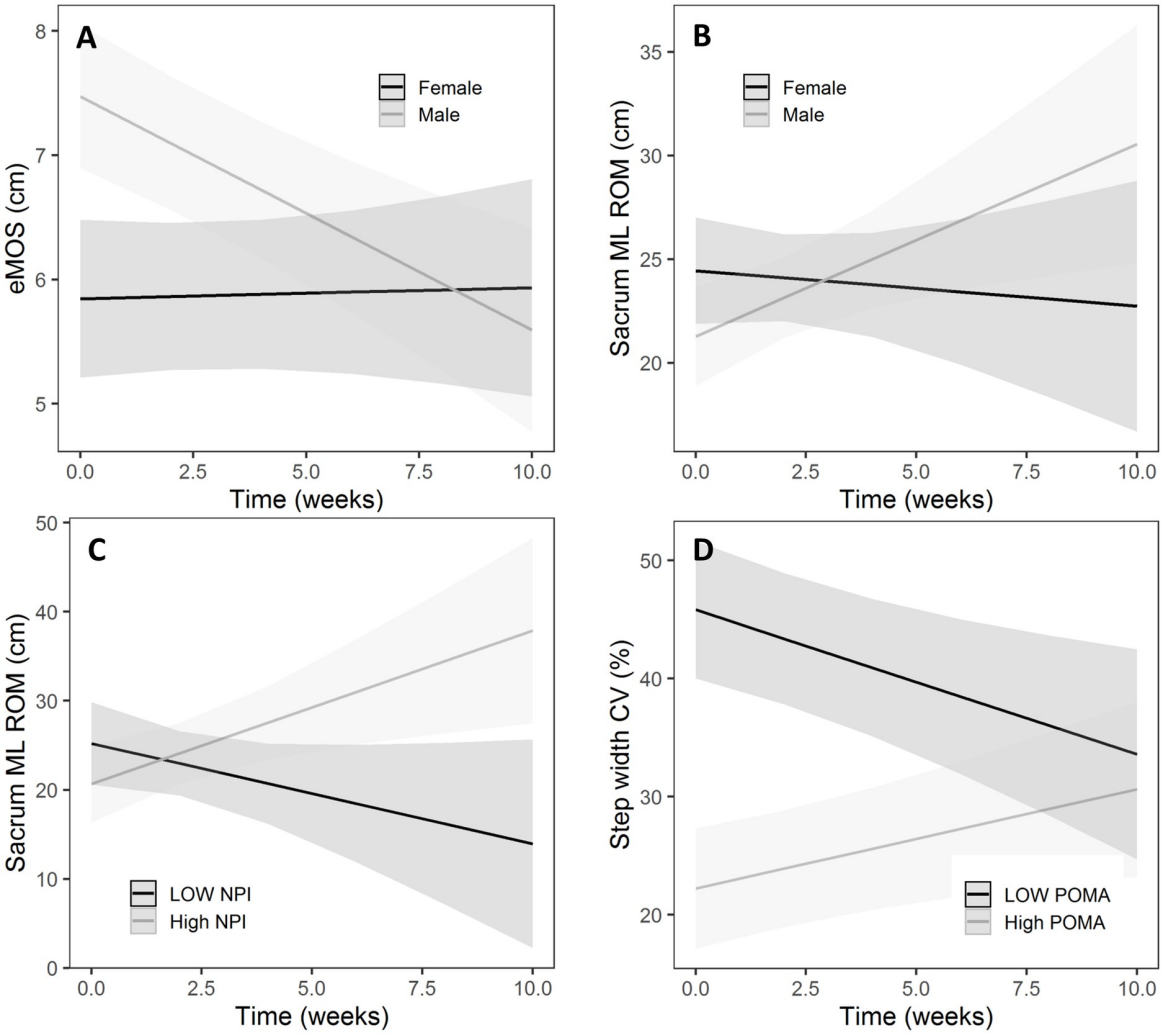
Plot of interaction effects using the conditional growth model. A) sex on eMOS, B) sex on sacrum ML ROM, C) NPI on sacrum ML ROM, and D) POMA-balance on step width CV. The light and dark gray areas are the 95% confidence intervals. See section 3.4 and Table 3 for further explanations. Note that for plotting purposes, the minimum and maximum values were used to dichotomize the continuous predictors (i.e. NPI and POMA-balance).

Clinical variables were added to the growth models to identify the relationship between these variables and change in gait over time. Over 10 weeks, the eMOS declined in men (Fig 2A) ~2 mm/week more than in women (p = 0.002, Table 3). Over this period, men’s sacrum ML ROM also increased (Fig 2B) on average 1.1 cm/week more than women (p = 0.02, Table 3). Moreover, while the sacrum ML ROM decreased for those with low NPI, it was increased for those with higher NPI (coefficient = 0.03 cm/week, p = 0.02, Table 3 and Fig 2C). Note that in Fig 2C, for plotting purposes, the minimum (= 4) and maximum (= 96) values of the NPI were used to draw the low and high NPI lines. Finally, those with higher POMA-balance initially had lower step width CV and increased it over time while those with low POMA-balance initially had a high step width CV and decreased it over 10 weeks (coefficient = 0.14%/week, Table 3 and Fig 2D). In Fig 2D, the low and high POMA lines correspond to the minimum (= 2) and maximum (= 16) values of the POMA. There was no effect of remaining clinical variables on gait changes over time. In particular, none of the variables of fall history, falls during the study, transfer to acute care hospital and psychotropic medication at baseline had statistically significant effects on the change of any gait measures over time (see the S1 File). Similar to the UGM, here also, we excluded the interaction of clinical variables with age or sex (e.g. Age x NPI or Sex x NPI) as predictors in the CGM models as there were no effects of the interaction of clinical variables with age or sex on the slope of the gait variable change over time.

**Table 3:** The effect of predictors on the slope of gait variables (change per week) using the conditional growth model.

Interactions	Coefficient	SD	CI	p-value
Sex on eMOS (cm/week)	-0.19	0.06	-0.31– -0.06	**0.002**
Sex on sacrum ML ROM (cm/week)	1.10	0.50	0.11–2.09	**0.02**
NPI on sacrum ML ROM (cm/week)	0.03	0.01	0.004–0.05	**0.02**
POMA-balance on step width CV (%/week)	0.14	0.05	0.04–0.25	**0.004**

eMOS = estimated margin of stability; CV = coefficient of variation; Sacrum ML ROM = sacrum range of motion in mediolateral direction; POMA = Tinetti Performance Oriented Mobility Assessment; NPI = neuropsychiatric inventory; SD = standard deviation; CI = 95% confidence interval; α = 0.05.

Here, only the significant interaction results are shown. See the S1 File for complete results.

## Discussion

This study makes use of data from a cohort of patients admitted to an inpatient psychogeriatric unit for treatment of BPSD, and monitored for changes in quantitative gait measures over the course of their admission, to explore potential measures of mobility change in this population. We found that eMOS (a measure of gait stability), step time, and step length decreased, and step time CV and step length CV increased over the 10-week period of observation, all indicating a deterioration of gait in this cohort of participants. We also found the following associations between the gait changes and clinical and demographic variables: gait stability with sex, sacrum lateral movement with neuropsychiatric changes, and step width variability with baseline balance performance but we did not find any relationship between gait changes over time and falls during the observation period.

Previous studies on longitudinal gait change in older adults have been focussed on community-dwelling older adults without dementia and were limited to measuring gait speed [25–27] or clinical measures of gait (e.g., POMA; [28]). Longitudinal gait assessments of older adults with dementia have been completed in studies with assessments several months to one year apart, over periods of four month to two years. These studies used both clinical measures of mobility and gait kinematic measures, although the gait measures were limited to step or stride spatiotemporal variables. In these studies, decline in mobility over time could be identified by decreased gait speed and step or stride length and increased stride length variability over time [29–31]. However, there is an important gap in understanding factors that contribute to these gait changes over time, and in characterizing changes in the stability of gait more directly.

While we found a decline in the lateral stability of gait (eMOS) over time in our participants, this was not associated with falls over the period of observation. This is in contrast to our previous findings that poor lateral stability at baseline is an important predictor of future falls and time to fall [12, 13, 18]. One possible explanation for the finding that declining stability was not associated with falls is that we also found evidence of changes in gait consistent with compensatory gait adaptations, such as decreased step time, and step length. Decreased step length and time in older adults is an indication of more cautious gait in this population [32, 33]. It is thus possible that as our participants’ stability declined over time, they acquired a more cautious walking pattern to avoid falls. However, whether these adaptive strategies can take place over a short period of time is not clear and requires further investigation. This is particularly important because some of our participants had as few as eight walks.

Another interesting finding was that poor performance on clinical balance assessments (POMA-balance) at the time of admission was correlated with high step width variability, and that step width variability decreased over time; this trend was reversed for those with good performance on clinical balance assessments at the time of admission (Fig 2D). Excessive step width variability is generally considered an indicator of a poor balance or higher fall risk [34] although contradictory findings have been reported [35, 36]. The fact that those with clinically recognized poor balance at admission had decreased step width variability over their admission could represent more active rehabilitation, or perhaps changes in treatment, such as cessation of medications contributing to poor balance. This reduction was not observed in those who had good balance at baseline, who instead experienced an increase in step width variability. It is also possible that balance as evaluated by POMA-balance is independent of the dynamic balance required for gait. Alternatively, another explanation could be the ‘regression to the mean’ phenomenon. Regression to the mean is a situation where, in repeated measurements on a subject, unusually large or small measurements or data points tend to be followed by data points that are closer to the mean of the data and it is due to random (or un-systematic) error with respect to the mean. In our context, a reduction (or increase) in step widths could be due to random errors in measurement or in stepping of the participants, where participants tend to step closer to the mean value after an unusually large (or small) step width.

Overall, we found a decline in gait over a period of 10 weeks in our study. Men exhibited more decline in gait stability, due to increased movements of their sacrum in the lateral direction (sacrum ML ROM), which placed their centre of mass closer to the boundaries of base of support. Biomechanically, these larger movements of the centre of mass could be compensated for by appropriate foot placement, although our participants failed to make these adaptations (as evidenced by no change of step width CV over time), which contributed to the observed decline in stability. Another possible factor associated with decreased markers of stability was the severity of neuropsychiatric symptoms (as indicated by NPI scores) at admission, as we found that sacrum ML ROM increased in those with high NPI at admission (Fig 2C). Our findings regarding the association between severe neuropsychiatric symptoms, male sex and more rapid decline in gait stability are supported by the findings of previous studies which found a correlation between severe behavioural symptoms (higher NPI) and lower scores on clinical balance measures [37]. Men also have more severe behavioural symptoms [38, 39] and tend to receive higher doses of psychotropic medications compared to women [40]. Men are also at higher risks of developing medication-related gait parkinsonism [41] which could lead to more severe gait disturbances. Thus, it is possible that, over the course of the admission, the men in this study and those with more severe behavioural symptoms were prescribed more psychotropic medications or medications at higher doses, which can adversely affect balance. However, our analysis of medication usage at baseline could not answer this question and a separate analysis with medication as a time-varying predictor and incorporating dose exposure is needed to reveal the effect of medication on gait changes. There is a need for further study to explore the short- and long-term effects of psychotropic medication on gait.

None of the remaining clinical measures (i.e., age, number of walks, fall history, falls during stay, hospitalization, medication at baseline) could explain the changes in any gait variables over time. In particular, we had expected falls during the inpatient stay to be correlated with gait change over time as falls can have a detrimental effect on mobility of older adults. One explanation could be that our analyses encompassed data from both before and after a fall (for fallers) and were not designed to detect a change in gait that occurred after the fall. A fall may cause participants to adopt a more cautious gait post-fall thus washing out the correlation of pre-fall gait and fall incident. Another explanation could be that adoption of a more cautious gait by the participant was a positive effect of medical and behavioral rehabilitation received during the hospital stay.

An important question to consider is whether the observed changes are clinically meaningful. Studies that have examined the minimum clinically important difference (MCID) or minimal detectable change (MDC) tend to focus on relatively healthy older adults, and less is known about clinically meaningful changes in people who are already at an advanced stage of cognitive impairment. In one study on older adults with cognitive decline [42], the MDC values were 1.6 cm/s for gait speed, 0.24 s for step time, 0.9 cm for step length, 0.1 cm for step width. In our study, we found an average 10 week change in gait speed of 4.2 cm/s (0.42 cm/s/week), step time of 0.04 s (0.004 s/week), step length of 4.8 cm (0.48 cm/week), and step width of 0.5 cm (0.05 cm/week). That some gait variables in our study had changes within the range of previously reported MDC suggests that clinically meaningful gait changes can occur over a hospitalization in older adults with advanced dementia, and demonstrates a potential value for frequent gait monitoring in this population. This detectable and clinically meaningful gait change over a hospitalization in older adults with advanced dementia shows the importance of frequent gait monitoring in this population.

An important limitation of this study is that we measured most of the clinical variables only at baseline when participants entered the study and did not track their change over time. We also did not track participants prospectively beyond the discharge to establish if mobility decline during the hospital stay was associated with falls or other adverse events related to mobility decline outside the study period. Moreover, we intentionally included only one predictor at a time in the CGM to keep the models and the interpretations simple as our aim in this study was primarily descriptive. Finally, there is the risk of measurement error: although our Kinect based system has been shown to be reliable [15–17], random measurement error can introduce bias. The repeated intensive longitudinal design helps to minimize random error through frequent and repeated measures, and our modelling was designed to take into account within-subject variability by incorporating random effects in the model. A related note is that, while the multilevel analysis takes into account the between-subject variability of the length of observations through random effects, the estimated slopes are the average over all participants and not necessarily applicable to each participant, especially those with very small number of observations and thus larger errors to the means (i.e. random effects).

## Conclusions

With repeated, longitudinal, quantitative measures of gait in a hospitalized older population with dementia over a 10-week period, we were able to detect decline in gait stability and the adoption of a more cautious walking pattern, while those with the most impaired gait on admission had improved gait. Men and those with more severe neuropsychiatric symptoms experienced the most decline in stability, although there was no relationship between decline in stability and falls during the observation period. Finally, while our approach quantifies changes in gait over time, it remains to be investigated what would represent a clinically significant decline.

## Supporting information

S1 File Longitudinal paper- supplementary material. (DOCX)
