# Supplementary material for: Gait changes over time in hospitalized older adults with advanced dementia: Predictors of mobility change
Source: PLoS One. 2021 Nov 17;16(11):e0259975. doi: 10.1371/journal.pone.0259975 (PMC8598066; doi:10.1371/journal.pone.0259975)
Supplement: S1 File — (DOCX) [file pone.0259975.s001.docx]

| **Supplementatry material** |
| --- |
| Paper: |
| Authors: |
| Journal: |
|  |
|  |
| Note: |
| The first two sheets are the results of uncondtional means and growth models respectively for all gait variables. |
| The third to last sheets are the results of conditional growth model for each gait variable with different predictors at each column. |
| See Table below for the details of the models |


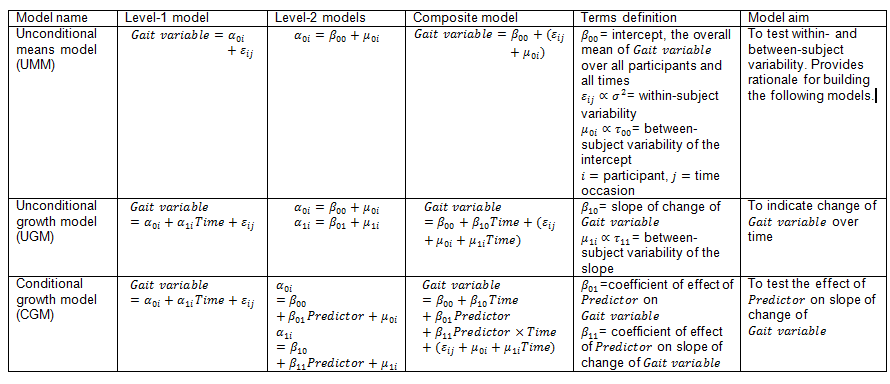


Unconditional means models

|  | **eMOS** | | | | **STEP TIME** | | | | **STEP LENGTH** | | | | **STEP TIME CV** | | | | **STEP LENGTH CV** | | | | **RMS SACR ML VELOCITY** | | | | **GAIT SPEED** | | | | **STEP WIDTH** | | | | **STEP WIDTH CV** | | | | **ROM SACR ML** | | | |
| --- | --- | --- | --- | --- | --- | --- | --- | --- | --- | --- | --- | --- | --- | --- | --- | --- | --- | --- | --- | --- | --- | --- | --- | --- | --- | --- | --- | --- | --- | --- | --- | --- | --- | --- | --- | --- | --- | --- | --- | --- |
|  | **Estimate** | **SD** | **CI** | **p-value** | **Estimate** | **SD** | **CI** | **p-value** | **Estimate** | **SD** | **CI** | **p-value** | **Estimate** | **SD** | **CI** | **p-value** | **Estimate** | **SD** | **CI** | **p-value** | **Estimate** | **SD** | **CI** | **p-value** | **Estimate** | **SD** | **CI** | **p-value** | **Estimate** | **SE/SD** | **CI** | **p-value** | **Estimate** | **SE/SD** | **CI** | **p-value** | **Estimate** | **SE/SD** | **CI** | **p-value** |
| **Fixed effects** | | | | | | | | | | | | | | | | | | | | | | | | | | | | | | | | | | | | | | | | |
| Intercept) | 6.4869 | 0.218 | 6.0597 – 6.9142 | **<0.001** | 0.6218 | 0.0098 | 0.6025 – 0.6411 | **<0.001** | 30.6846 | 0.9744 | 28.7747 – 32.5944 | **<0.001** | 22.2729 | 1.187 | 19.9464 – 24.5995 | **<0.001** | 26.8394 | 1.3241 | 24.2442 – 29.4345 | **<0.001** | 17.9013 | 0.3613 | 17.1932 – 18.6094 | **<0.001** | 49.6145 | 1.6688 | 46.3437 – 52.8852 | **<0.001** | 16.7741 | 0.4058 | 15.9788 – 17.5694 | **<0.001** | 32.9002 | 1.2423 | 30.4653 – 35.3351 | **<0.001** | 23.829 | 0.7457 | 22.3675 – 25.2905 | **<0.001** |
| PREDICTOR |  |  |  |  |  |  |  |  |  |  |  |  |  |  |  |  |  |  |  |  |  |  |  |  |  |  |  |  |  |  |  |  |  |  |  |  |  |  |  |  |
| TIME |  |  |  |  |  |  |  |  |  |  |  |  |  |  |  |  |  |  |  |  |  |  |  |  |  |  |  |  |  |  |  |  |  |  |  |  |  |  |  |  |
| PREDICTOR * TIME |  |  |  |  |  |  |  |  |  |  |  |  |  |  |  |  |  |  |  |  |  |  |  |  |  |  |  |  |  |  |  |  |  |  |  |  |  |  |  |  |
| **Random effects** | | | | | | | | | | | | | | | | | | | | | | | | | | | | | | | | | | | | | | | | |
| σ2 (within-subject) | 5.938 | 2.437 | 5.694 - 6.196 |  | 0.014 | 0.12 | 0.014 - 0.015 |  | 53.665 | 7.326 | 51.461 - 55.998 |  | 440.103 | 20.979 | 421.996 - 459.262 |  | 485.118 | 22.025 | 465.174 - 506.22 |  | 60.268 | 7.763 | 57.79 - 62.889 |  | 155.798 | 12.482 | 149.397 - 162.57 |  | 12.437 | 3.527 | 11.926 - 12.978 |  | 466.336 | 21.595 | 447.176 - 486.607 |  | 328.8 | 18.133 | 315.266 - 343.12 |  |
| τ00:id (between-subject) | 2.428 | 1.558 | 1.662 - 3.7 |  | 0.005 | 0.07 | 0.003 - 0.008 |  | 49.986 | 7.07 | 34.686 - 75.37 |  | 66.526 | 8.156 | 41.725 - 108.976 |  | 84.008 | 9.166 | 54.537 - 133.691 |  | 5.794 | 2.407 | 3.615 - 9.492 |  | 146.639 | 12.109 | 101.665 - 221.257 |  | 8.596 | 2.932 | 5.956 - 12.975 |  | 73.172 | 8.554 | 48.337 - 114.645 |  | 23.4 | 4.837 | 13.423 - 40.799 |  |
| τ11: TIME (between-subject) |  |  |  |  |  |  |  |  |  |  |  |  |  |  |  |  |  |  |  |  |  |  |  |  |  |  |  |  |  |  |  |  |  |  |  |  |  |  |  |  |
| **Model performance** | | | | | | | | | | | | | | | | | | | | | | | | | | | | | | | | | | | | | | | | |
| Deviance |  |  | 20305.61491 |  |  |  | -5979.194464 |  |  |  | 29941.81155 |  |  |  | 39020.17095 |  |  |  | 39451.03634 |  |  |  | 30334.80486 |  |  |  | 34587.08103 |  |  |  | 23554.39392 |  |  |  | 39274.2533 |  |  |  | 37715.78961 |  |
| AIC |  |  | 20311.61491 |  |  |  | -5973.194464 |  |  |  | 29947.81155 |  |  |  | 39026.17095 |  |  |  | 39457.03634 |  |  |  | 30340.80486 |  |  |  | 34593.08103 |  |  |  | 23560.39392 |  |  |  | 39280.2533 |  |  |  | 37721.78961 |  |
| log-Likelihood |  |  | -10152.80746 |  |  |  | 2989.597232 |  |  |  | -14970.90578 |  |  |  | -19510.08548 |  |  |  | -19725.51817 |  |  |  | -15167.40243 |  |  |  | -17293.54051 |  |  |  | -11777.19696 |  |  |  | -19637.1267 |  |  |  | -18857.89481 |  |

Unconditional growth models

|  | **eMOS** | | | | **STEP TIME** | | | | **STEP LENGTH** | | | | **STEP TIME CV** | | | | **STEP LENGTH CV** | | | | **RMS SACR ML VELOCITY** | | | | **GAIT SPEED** | | | | **STEP WIDTH** | | | | **STEP WIDTH CV** | | | | **ROM SACR ML** | | | |
| --- | --- | --- | --- | --- | --- | --- | --- | --- | --- | --- | --- | --- | --- | --- | --- | --- | --- | --- | --- | --- | --- | --- | --- | --- | --- | --- | --- | --- | --- | --- | --- | --- | --- | --- | --- | --- | --- | --- | --- | --- |
|  | **Estimate** | **SD** | **CI** | **p-value** | **Estimate** | **SD** | **CI** | **p-value** | **Estimate** | **SD** | **CI** | **p-value** | **Estimate** | **SD** | **CI** | **p-value** | **Estimate** | **SD** | **CI** | **p-value** | **Estimate** | **SD** | **CI** | **p-value** | **Estimate** | **SD** | **CI** | **p-value** | **Estimate** | **SD** | **CI** | **p-value** | **Estimate** | **SD** | **CI** | **p-value** | **Estimate** | **SD** | **CI** | **p-value** |
| **Fixed effects** | | | | | | | | | | | | | | | | | | | | | | | | | | | | | | | | | | | | | | | | |
| Intercept | 6.7403 | 0.2466 | 6.2569 – 7.2237 | **<0.001** | 0.6339 | 0.0104 | 0.6137 – 0.6542 | **<0.001** | 31.9251 | 1.0189 | 29.9280 – 33.9221 | **<0.001** | 19.1241 | 1.032 | 17.1015 – 21.1467 | **<0.001** | 23.3914 | 1.3139 | 20.8162 – 25.9667 | **<0.001** | 17.3836 | 0.3629 | 16.6723 – 18.0948 | **<0.001** | 50.6159 | 1.7853 | 47.1167 – 54.1151 | **<0.001** | 16.5996 | 0.4833 | 15.6522 – 17.5469 | **<0.001** | 32.8999 | 1.4108 | 30.1348 – 35.6650 | **<0.001** | 22.7633 | 0.9404 | 20.9203 – 24.6064 | **<0.001** |
| PREDICTOR |  |  |  |  |  |  |  |  |  |  |  |  |  |  |  |  |  |  |  |  |  |  |  |  |  |  |  |  |  |  |  |  |  |  |  |  |  |  |  |  |
| TIME | -0.099 | 0.0337 | -0.1650 – -0.0330 | **0.003** | -0.0048 | 0.0016 | -0.0079 – -0.0017 | **0.002** | -0.4883 | 0.1299 | -0.7430 – -0.2336 | **<0.001** | 1.2254 | 0.3446 | 0.5500 – 1.9008 | **<0.001** | 1.2603 | 0.3704 | 0.5343 – 1.9863 | **0.001** | 0.1941 | 0.0995 | -0.0008 – 0.3891 | 0.051 | -0.4297 | 0.2408 | -0.9016 – 0.0422 | 0.074 | 0.0622 | 0.0623 | -0.0599 – 0.1844 | 0.318 | -0.0052 | 0.2034 | -0.4039 – 0.3936 | 0.98 | 0.4021 | 0.2722 | -0.1313 – 0.9356 | 0.14 |
| PREDICTOR * TIME |  |  |  |  |  |  |  |  |  |  |  |  |  |  |  |  |  |  |  |  |  |  |  |  |  |  |  |  |  |  |  |  |  |  |  |  |  |  |  |  |
| **Random effects** | | | | | | | | | | | | | | | | | | | | | | | | | | | | | | | | | | | | | | | | |
| σ2 (within-subject) | 5.83E+00 | 2.414 | 5.587 - 6.0832 |  | 1.40E-02 | 0.118 | 0.013 - 0.0146 |  | 5.11E+01 | 7.146 | 48.952 - 53.2914 |  | 4.25E+02 | 20.622 | 407.716 - 443.8818 |  | 4.68E+02 | 21.632 | 448.581 - 488.4097 |  | ####### | 7.706 | 56.925 - 61.9753 |  | 149.0318 | 12.208 | 142.877 - 155.5455 |  | 12.0657 | 3.474 | 11.567 - 12.5935 |  | 464.801 | 21.559 | 445.614 - 485.1019 |  | 322.7593 | 17.966 | 309.398 - 336.9011 |  |
| τ00:id (between-subject) | 2.9628 | 1.721 | 1.975 - 4.606 |  | 0.005 | 0.071 | 0.003 - 0.008 |  | 53.0459 | 7.283 | 36.275 - 80.8937 |  | 36.0392 | 6.003 | 19.476 - 65.2298 |  | 67.9135 | 8.241 | 38.694 - 118.8989 |  | 4.2291 | 2.056 | 1.955 - 8.3007 |  | 163.163 | 12.774 | 111.909 - 248.2431 |  | 11.9078 | 3.451 | 8.12 - 18.1985 |  | 85.1706 | 9.229 | 53.795 - 138.1336 |  | 30.9556 | 5.564 | 16.374 - 56.4192 |  |
| τ11: TIME (between-subject) | 0.0289 | 0.17 | 0.011 - 0.0624 |  | 1.00E-04 | 0.008 | 0 - 1e-04 |  | 5.54E-01 | 0.745 | 0.31 - 0.9889 |  | 3.71E+00 | 1.926 | 1.865 - 7.1137 |  | 4.29E+00 | 2.071 | 2.239 - 8.0007 |  | 2.19E-01 | 0.468 | 0.082 - 0.4878 |  | 2.0452 | 1.43 | 1.171 - 3.5799 |  | 0.1328 | 0.364 | 0.07 - 0.2418 |  | 0.4243 | 0.651 | 0 - 1.4986 |  | 2.1059 | 1.451 | 0.893 - 4.3591 |  |
| **Model performance** | | | | | | | | | | | | | | | | | | | | | | | | | | | | | | | | | | | | | | | | |
| Deviance | 20263.48 |  |  |  | -6029.82 |  |  |  | 29788.59 |  |  |  | 38912.12 |  |  |  | 39344.23 |  |  |  | 30296.7 |  |  |  | 34462.1 |  |  |  | 23481 |  |  |  | 39270.6 |  |  |  | 37680.3 |  |  |  |
| AIC | 20275.48 |  |  |  | -6017.82 |  |  |  | 29800.59 |  |  |  | 38924.12 |  |  |  | 39356.23 |  |  |  | 30308.7 |  |  |  | 34474.1 |  |  |  | 23493 |  |  |  | 39282.6 |  |  |  | 37692.3 |  |  |  |
| log-Likelihood | -10131.7 |  |  |  | 3014.91 |  |  |  | -14894.3 |  |  |  | -19456.1 |  |  |  | -19672.1 |  |  |  | -15148 |  |  |  | -17231 |  |  |  | -11740 |  |  |  | -19635 |  |  |  | -18840 |  |  |  |

**Conditional growth model - eMOS**

| **Predictors** | **Age** | | | | **Sex** | | | | **POMAB** | | | | **NPI** | | | | **Fall history** | | | | **No. of walks** | | | | **Falls during stay** | | | | **Hospitalization** | | | | **Antipsychotics** | | | | **Benzodiazepines** | | | | **Antidepressants** | | | |
| --- | --- | --- | --- | --- | --- | --- | --- | --- | --- | --- | --- | --- | --- | --- | --- | --- | --- | --- | --- | --- | --- | --- | --- | --- | --- | --- | --- | --- | --- | --- | --- | --- | --- | --- | --- | --- | --- | --- | --- | --- | --- | --- | --- | --- |
|  | **Estimate** | **SD** | **CI** | **p-value** | **Estimate** | **SD** | **CI** | **p-value** | **Estimate** | **SD** | **CI** | **p-value** | **Estimate** | **SD** | **CI** | **p-value** | **Estimate** | **SD** | **CI** | **p-value** | **Estimate** | **SD** | **CI** | **p-value** | **Estimate** | **SD** | **CI** | **p-value** | **Estimate** | **SD** | **CI** | **p-value** | **Estimate** | **SD** | **CI** | **p-value** | **Estimate** | **SD** | **CI** | **p-value** | **Estimate** | **SD** | **CI** | **p-value** |
| **Fixed effects** | | | | | | | | | | | | | | | | | | | | | | | | | | | | | | | | | | | | | | | | | | | | |
| Intercept) | 13.3181 | 2.2607 | 8.8872 – 17.7491 | **<0.001** | 5.845 | 0.3245 | 5.2089 – 6.4811 | **<0.001** | 4.6281 | 0.7291 | 3.1991 – 6.0571 | **<0.001** | 7.1801 | 0.6645 | 5.8777 – 8.4825 | **<0.001** | 6.6536 | 0.2932 | 6.0790 – 7.2282 | **<0.001** | 6.8876 | 0.3818 | 6.1393 – 7.6358 | **<0.001** | 6.7949 | 0.246 | 6.3128 – 7.2771 | **<0.001** | 6.5514 | 0.2801 | 6.0024 – 7.1005 | **<0.001** | 6.9704 | 0.5657 | 5.8616 – 8.0792 | **<0.001** | 6.5919 | 0.2994 | 6.0051 – 7.1787 | **<0.001** | 6.7544 | 0.3745 | 6.0203 – 7.4885 | **<0.001** |
| PREDICTOR | -0.0861 | 0.0295 | -0.1438 – -0.0283 | **0.003** | 1.6379 | 0.438 | 0.7793 – 2.4964 | **<0.001** | 0.2229 | 0.0729 | 0.0800 – 0.3657 | **0.002** | -0.0084 | 0.0118 | -0.0316 – 0.0147 | 0.475 | 0.2958 | 0.5373 | -0.7572 – 1.3488 | 0.582 | -0.0014 | 0.0035 | -0.0082 – 0.0055 | 0.693 | -0.2225 | 0.1594 | -0.5349 – 0.0898 | 0.163 | -0.1097 | 0.0389 | -0.1859 – -0.0335 | **0.005** | -0.125 | 0.0706 | -0.2633 – 0.0134 | 0.077 | -0.0903 | 0.0416 | -0.1719 – -0.0088 | **0.03** | -0.1185 | 0.0481 | -0.2128 – -0.0242 | **0.014** |
| TIME | -0.2474 | 0.3274 | -0.8890 – 0.3943 | 0.45 | 0.009 | 0.0453 | -0.0798 – 0.0978 | 0.842 | 0.0015 | 0.1063 | -0.2069 – 0.2098 | 0.989 | -0.0939 | 0.0976 | -0.2851 – 0.0974 | 0.336 | -0.115 | 0.0407 | -0.1947 – -0.0353 | **0.005** | -0.1755 | 0.0527 | -0.2788 – -0.0722 | **0.001** | -0.0921 | 0.0364 | -0.1634 – -0.0208 | **0.011** | 0.7169 | 0.5566 | -0.3739 – 1.8078 | 0.198 | -0.291 | 0.6278 | -1.5215 – 0.9394 | 0.643 | 0.429 | 0.5178 | -0.5859 – 1.4440 | 0.407 | -0.0412 | 0.4959 | -1.0131 – 0.9306 | 0.934 |
| PREDICTOR * TIME | 0.0019 | 0.0043 | -0.0065 – 0.0103 | 0.652 | -0.2017 | 0.0623 | -0.3238 – -0.0795 | **0.001** | -0.0104 | 0.0104 | -0.0309 – 0.0101 | 0.319 | -0.0001 | 0.0017 | -0.0035 – 0.0033 | 0.96 | 0.0457 | 0.0706 | -0.0926 – 0.1840 | 0.517 | 0.0007 | 0.0004 | -0.0001 – 0.0015 | 0.07 | 0.0221 | 0.0198 | -0.0167 – 0.0609 | 0.265 | 0.0421 | 0.0745 | -0.1039 – 0.1881 | 0.572 | 0.036 | 0.0801 | -0.1209 – 0.1929 | 0.65 | -0.0189 | 0.0692 | -0.1545 – 0.1167 | 0.785 | 0.0425 | 0.067 | -0.0888 – 0.1738 | 0.526 |
| **Random effects** | | | | | | | | | | | | | | | | | | | | | | | | | | | | | | | | | | | | | | | | | | | | |
| σ2 (within-subject) | 5.8286 | 2.414 | 5.588 - 6.0836 |  | 5.8261 | 2.414 | 5.585 - 6.0807 |  | 5.8283 | 2.414 | 5.587 - 6.0833 |  | 5.8281 | 2.414 | 5.587 - 6.0831 |  | 5.8293 | 2.414 | 5.588 - 6.0844 |  | 5.8327 | 2.415 | 5.591 - 6.0881 |  | 5.8275 | 2.414 | 5.587 - 6.0825 |  | 5.8236 | 2.413 | 5.583 - 6.0784 |  | 5.8237 | 2.413 | 5.586 - 6.0554 |  | 5.8241 | 2.413 | 5.577 - 6.0901 |  | 5.8226 | 2.413 | 5.569 - 6.0528 |  |
| τ00:id (between-subject) | 2.5047 | 1.583 | 1.66 - 3.9113 |  | 2.2526 | 1.501 | 1.474 - 3.5504 |  | 2.4826 | 1.576 | 1.641 - 3.8877 |  | 2.9327 | 1.712 | 1.953 - 4.5629 |  | 2.9379 | 1.714 | 1.959 - 4.5678 |  | 2.8916 | 1.7 | 1.924 - 4.5022 |  | 2.8525 | 1.689 | 1.891 - 4.4566 |  | 2.8437 | 1.686 | 1.889 - 4.4332 |  | 2.9266 | 1.711 | 1.759 - 4.2041 |  | 2.9013 | 1.703 | 1.702 - 4.2507 |  | 2.9297 | 1.712 | 1.707 - 3.9985 |  |
| τ11: TIME (between-subject) | 2.82E-02 | 0.168 | 0.011 - 0.0608 |  | 2.11E-02 | 0.145 | 0.008 - 0.0472 |  | 2.76E-02 | 0.166 | 0.01 - 0.0618 |  | 2.91E-02 | 0.171 | 0.011 - 0.0628 |  | 2.77E-02 | 0.167 | 0.01 - 0.0608 |  | 2.20E-02 | 0.148 | 0.006 - 0.0528 |  | 3.14E-02 | 0.177 | 0.012 - 0.0679 |  | 2.76E-02 | 0.166 | 0.01 - 0.0597 |  | 2.77E-02 | 0.167 | 0.007 - 0.0468 |  | 2.76E-02 | 0.166 | 0.007 - 0.0461 |  | 2.82E-02 | 0.168 | 0.009 - 0.0494 |  |
| **Model performance** | | | | | | | | | | | | | | | | | | | | | | | | | | | | | | | | | | | | | | | | | | | | |
| Deviance | 20254.25 |  |  |  | 20247.9 |  |  |  | 20254.57418 |  |  |  | 20262.8 |  |  |  | 20262 |  |  |  | 20260.3 |  |  |  | 20261.6 |  |  |  | 20255.9 |  |  |  | 20259.3 |  |  |  | 20258.8 |  |  |  | 20259.1 |  |  |  |
| AIC | 20270.25 |  |  |  | 20263.9 |  |  |  | 20270.57418 |  |  |  | 20278.8 |  |  |  | 20278 |  |  |  | 20276.3 |  |  |  | 20277.6 |  |  |  | 20271.9 |  |  |  | 20275.3 |  |  |  | 20274.8 |  |  |  | 20275.1 |  |  |  |
| log-Likelihood | -10127.1 |  |  |  | -10124 |  |  |  | -10127.28709 |  |  |  | -10131 |  |  |  | -10131 |  |  |  | -10130 |  |  |  | -10131 |  |  |  | -10128 |  |  |  | -10130 |  |  |  | -10129 |  |  |  | -10130 |  |  |  |

**Conditional growth model – step time**

| **Predictors** | **Age** | | | | **Sex** | | | | **POMAB** | | | | **NPI** | | | | **Fall history** | | | | **No. of walks** | | | | **Falls during stay** | | | | **Hospitalization** | | | | **Antipsychotics** | | | | **Benzodiazepines** | | | | **Antidepressants** | | | |
| --- | --- | --- | --- | --- | --- | --- | --- | --- | --- | --- | --- | --- | --- | --- | --- | --- | --- | --- | --- | --- | --- | --- | --- | --- | --- | --- | --- | --- | --- | --- | --- | --- | --- | --- | --- | --- | --- | --- | --- | --- | --- | --- | --- | --- |
|  | **Estimate** | **SD** | **CI** | **p-value** | **Estimate** | **SD** | **CI** | **p-value** | **Estimate** | **SD** | **CI** | **p-value** | **Estimate** | **SD** | **CI** | **p-value** | **Estimate** | **SD** | **CI** | **p-value** | **Estimate** | **SD** | **CI** | **p-value** | **Estimate** | **SD** | **CI** | **p-value** | **Estimate** | **SD** | **CI** | **p-value** | **Estimate** | **SD** | **CI** | **p-value** | **Estimate** | **SD** | **CI** | **p-value** | **Estimate** | **SD** | **CI** | **p-value** |
| **Fixed effects** | | | | | | | | | | | | | | | | | | | | | | | | | | | | | | | | | | | | | | | | | | | | |
| Intercept) | 0.7135 | 0.1017 | 0.5142 – 0.9128 | **<0.001** | 0.6113 | 0.0149 | 0.5822 – 0.6404 | **<0.001** | 0.5608 | 0.0314 | 0.4994 – 0.6223 | **<0.001** | 0.6368 | 0.028 | 0.5819 – 0.6917 | **<0.001** | 0.6298 | 0.0123 | 0.6057 – 0.6540 | **<0.001** | 0.6358 | 0.0163 | 0.6039 – 0.6678 | **<0.001** | 0.6366 | 0.0104 | 0.6162 – 0.6570 | **<0.001** | 0.6288 | 0.0119 | 0.6055 – 0.6521 | **<0.001** | 0.5902 | 0.0228 | 0.5455 – 0.6349 | **<0.001** | 0.6322 | 0.0127 | 0.6073 – 0.6571 | **<0.001** | 0.619 | 0.0155 | 0.5886 – 0.6495 | **<0.001** |
| PREDICTOR | -0.001 | 0.0013 | -0.0036 – 0.0016 | 0.432 | 0.0412 | 0.0201 | 0.0019 – 0.0806 | **0.04** | 0.0077 | 0.0031 | 0.0015 – 0.0138 | **0.014** | -0.0001 | 0.0005 | -0.0010 – 0.0009 | 0.913 | 0.0141 | 0.0226 | -0.0302 – 0.0584 | 0.533 | 0 | 0.0001 | -0.0003 – 0.0003 | 0.915 | -0.0092 | 0.0076 | -0.0241 – 0.0058 | 0.228 | -0.0051 | 0.0018 | -0.0087 – -0.0015 | **0.005** | -0.0008 | 0.0032 | -0.0072 – 0.0055 | 0.802 | -0.0058 | 0.002 | -0.0097 – -0.0020 | **0.003** | -0.004 | 0.0022 | -0.0084 – 0.0004 | 0.077 |
| TIME | 0.0005 | 0.0155 | -0.0298 – 0.0308 | 0.974 | -0.0033 | 0.0023 | -0.0078 – 0.0012 | 0.155 | -0.0008 | 0.005 | -0.0105 – 0.0090 | 0.879 | -0.0043 | 0.0046 | -0.0133 – 0.0047 | 0.348 | -0.0057 | 0.0019 | -0.0094 – -0.0019 | **0.003** | -0.006 | 0.0026 | -0.0112 – -0.0008 | **0.023** | -0.005 | 0.0016 | -0.0082 – -0.0017 | **0.003** | 0.0201 | 0.0237 | -0.0264 – 0.0665 | 0.397 | 0.0539 | 0.0253 | 0.0044 – 0.1035 | **0.033** | 0.0056 | 0.022 | -0.0376 – 0.0487 | 0.801 | 0.0262 | 0.0206 | -0.0142 – 0.0666 | 0.204 |
| PREDICTOR * TIME | -0.0001 | 0.0002 | -0.0005 – 0.0003 | 0.731 | -0.0028 | 0.0032 | -0.0090 – 0.0034 | 0.375 | -0.0004 | 0.0005 | -0.0014 – 0.0005 | 0.4 | 0 | 0.0001 | -0.0002 – 0.0002 | 0.915 | 0.0024 | 0.0033 | -0.0040 – 0.0089 | 0.461 | 0 | 0 | -0.0000 – 0.0000 | 0.569 | 0.0013 | 0.0009 | -0.0005 – 0.0031 | 0.158 | 0.0012 | 0.0035 | -0.0057 – 0.0081 | 0.735 | -0.005 | 0.0037 | -0.0122 – 0.0022 | 0.173 | 0.0029 | 0.0033 | -0.0035 – 0.0093 | 0.374 | -0.0015 | 0.0031 | -0.0077 – 0.0046 | 0.627 |
| **Random effects** | | | | | | | | | | | | | | | | | | | | | | | | | | | | | | | | | | | | | | | | | | | | |
| σ2 (within-subject) | 0.014 | 0.118 | 0.013 - 0.0146 |  | 0.014 | 0.118 | 0.013 - 0.0146 |  | 0.014 | 0.118 | 0.013 - 0.0146 |  | 0.014 | 0.118 | 0.013 - 0.0146 |  | 0.014 | 0.118 | 0.013 - 0.0146 |  | 0.014 | 0.118 | 0.013 - 0.0146 |  | 0.014 | 0.118 | 0.013 - 0.0146 |  | 0.014 | 0.118 | 0.013 - 0.0146 |  | 5.8237 | 2.413 | 0.013 - 0.0146 |  | 0.014 | 0.118 | 0.013 - 0.0146 |  | 0.014 | 0.118 | 0.013 - 0.0146 |  |
| τ00:id (between-subject) | 0.005 | 0.07 | 0.003 - 0.0079 |  | 0.0046 | 0.068 | 0.003 - 0.0074 |  | 0.0044 | 0.067 | 0.003 - 0.0071 |  | 0.005 | 0.071 | 0.003 - 0.008 |  | 0.005 | 0.071 | 0.003 - 0.008 |  | 0.005 | 0.071 | 0.003 - 0.008 |  | 0.0049 | 0.07 | 0.003 - 0.0078 |  | 0.005 | 0.07 | 0.003 - 0.0079 |  | 2.9266 | 1.711 | 0.003 - 0.0064 |  | 0.0051 | 0.071 | 0.003 - 0.0069 |  | 0.0049 | 0.07 | 0.003 - 0.0071 |  |
| τ11: TIME (between-subject) | 1.00E-04 | 0.008 | 0 - 1e-04 |  | 1.00E-04 | 0.008 | 0 - 1e-04 |  | 1.00E-04 | 0.007 | 0 - 1e-04 |  | 1.00E-04 | 0.008 | 0 - 1e-04 |  | 1.00E-04 | 0.007 | 0 - 1e-04 |  | 1.00E-04 | 0.007 | 0 - 1e-04 |  | 1.00E-04 | 0.007 | 0 - 1e-04 |  | 1.00E-04 | 0.007 | 0 - 1e-04 |  | 2.77E-02 | 0.167 | 0 - 1e-04 |  | 1.00E-04 | 0.008 | 0 - 1e-04 |  | 1.00E-04 | 0.007 | 0 - 1e-04 |  |
| **Model performance** | | | | | | | | | | | | | | | | | | | | | | | | | | | | | | | | | | | | | | | | | | | | |
| Deviance | -6030.8495 |  |  |  | -6033.978399 |  |  |  | -6035.531 |  |  |  | -6029.8548 |  |  |  | -6031.1733 |  |  |  | -6030.1352 |  |  |  | -6031.8 |  |  |  | -6028.6 |  |  |  | -6032.3 |  |  |  | -6028.5 |  |  |  | -6029 |  |  |  |
| AIC | -6014.8495 |  |  |  | -6017.978399 |  |  |  | -6019.531 |  |  |  | -6013.8548 |  |  |  | -6015.1733 |  |  |  | -6014.1352 |  |  |  | -6015.8 |  |  |  | -6012.6 |  |  |  | -6016.3 |  |  |  | -6012.5 |  |  |  | -6013 |  |  |  |
| log-Likelihood | 3015.42473 |  |  |  | 3016.9892 |  |  |  | 3017.76548 |  |  |  | 3014.9274 |  |  |  | 3015.58664 |  |  |  | 3015.06758 |  |  |  | 3015.88 |  |  |  | 3014.29 |  |  |  | 3016.17 |  |  |  | 3014.27 |  |  |  | 3014.51 |  |  |  |

**Conditional growth model – step length**

| **Predictors** | **Age** | | | | **Sex** | | | | **POMAB** | | | | **NPI** | | | | **Fall history** | | | | **No. of walks** | | | | **Falls during stay** | | | | **Hospitalization** | | | | **Antipsychotics** | | | | **Benzodiazepines** | | | | **Antidepressants** | | | |
| --- | --- | --- | --- | --- | --- | --- | --- | --- | --- | --- | --- | --- | --- | --- | --- | --- | --- | --- | --- | --- | --- | --- | --- | --- | --- | --- | --- | --- | --- | --- | --- | --- | --- | --- | --- | --- | --- | --- | --- | --- | --- | --- | --- | --- |
|  | **Estimate** | **SD** | **CI** | **p-value** | **Estimate** | **SD** | **CI** | **p-value** | **Estimate** | **SD** | **CI** | **p-value** | **Estimate** | **SD** | **CI** | **p-value** | **Estimate** | **SD** | **CI** | **p-value** | **Estimate** | **SD** | **CI** | **p-value** | **Estimate** | **SD** | **CI** | **p-value** | **Estimate** | **SD** | **CI** | **p-value** | **Estimate** | **SD** | **CI** | **p-value** | **Estimate** | **SD** | **CI** | **p-value** | **Estimate** | **SD** | **CI** | **p-value** |
| **Fixed effects** |  |  |  |  |  |  |  |  |  |  |  |  |  |  |  |  |  |  |  |  |  |  |  |  |  |  |  |  |  |  |  |  |  |  |  |  |  |  |  |  |  |  |  |  |
| Intercept) | 66.4815 | 8.8678 | 49.1009 – 83.8621 | **<0.001** | 32.8507 | 1.5135 | 29.8842 – 35.8172 | **<0.001** | 30.377 | 3.253 | 24.0013 – 36.7526 | **<0.001** | 28.7368 | 2.7157 | 23.4142 – 34.0594 | **<0.001** | 32.4199 | 1.2092 | 30.0500 – 34.7898 | **<0.001** | 30.0462 | 1.5481 | 27.0119 – 33.0804 | **<0.001** | 31.7456 | 1.0374 | 29.7124 – 33.7788 | **<0.001** | 31.6117 | 1.1809 | 29.2972 – 33.9262 | **<0.001** | 32.0965 | 2.3586 | 27.4736 – 36.7193 | **<0.001** | 31.4169 | 1.242 | 28.9826 – 33.8513 | **<0.001** | 31.6267 | 1.5556 | 28.5778 – 34.6756 | **<0.001** |
| PREDICTOR | -0.4521 | 0.1155 | -0.6785 – -0.2257 | **<0.001** | -1.6515 | 2.037 | -5.6439 – 2.3410 | 0.418 | 0.1611 | 0.3262 | -0.4783 – 0.8004 | 0.621 | 0.0611 | 0.0483 | -0.0336 – 0.1558 | 0.206 | -1.6749 | 2.2184 | -6.0230 – 2.6731 | 0.45 | 0.0244 | 0.0144 | -0.0038 – 0.0525 | 0.09 | 0.8127 | 0.5413 | -0.2482 – 1.8736 | 0.133 | -0.3816 | 0.1498 | -0.6751 – -0.0880 | **0.011** | -0.6993 | 0.2749 | -1.2380 – -0.1606 | **0.011** | -0.4542 | 0.1603 | -0.7684 – -0.1399 | **0.005** | -0.5285 | 0.1894 | -0.8998 – -0.1572 | **0.005** |
| TIME | -0.7184 | 1.2639 | -3.1956 – 1.7587 | 0.57 | -0.2788 | 0.1855 | -0.6423 – 0.0847 | 0.133 | 0.0994 | 0.4025 | -0.6895 – 0.8883 | 0.805 | -0.4905 | 0.3665 | -1.2089 – 0.2279 | 0.181 | -0.4679 | 0.1582 | -0.7780 – -0.1579 | **0.003** | -0.8046 | 0.2045 | -1.2055 – -0.4038 | **<0.001** | -0.5289 | 0.1344 | -0.7923 – -0.2655 | **<0.001** | 1.219 | 2.3328 | -3.3533 – 5.7913 | 0.601 | -0.2318 | 2.6159 | -5.3588 – 4.8953 | 0.929 | 1.5085 | 2.15 | -2.7054 – 5.7224 | 0.483 | 0.5013 | 2.0563 | -3.5291 – 4.5316 | 0.807 |
| PREDICTOR * TIME | 0.003 | 0.0165 | -0.0294 – 0.0353 | 0.857 | -0.3973 | 0.2549 | -0.8968 – 0.1022 | 0.119 | -0.0616 | 0.04 | -0.1400 – 0.0167 | 0.123 | 0 | 0.0065 | -0.0127 – 0.0127 | 0.998 | -0.0566 | 0.2765 | -0.5986 – 0.4854 | 0.838 | 0.003 | 0.0016 | -0.0002 – 0.0062 | 0.066 | -0.0718 | 0.064 | -0.1972 – 0.0536 | 0.262 | -0.3889 | 0.2887 | -0.9548 – 0.1770 | 0.178 | 0.2739 | 0.3112 | -0.3360 – 0.8838 | 0.379 | -0.0936 | 0.2726 | -0.6279 – 0.4406 | 0.731 | 0.0806 | 0.2598 | -0.4286 – 0.5899 | 0.756 |
| **Random effects** |  |  |  |  |  |  |  |  |  |  |  |  |  |  |  |  |  |  |  |  |  |  |  |  |  |  |  |  |  |  |  |  |  |  |  |  |  |  |  |  |  |  |  |  |
| σ2 (within-subject) | 51.0629 | 7.146 | 48.954 - 53.2943 |  | 51.055 | 7.145 | 48.947 - 53.2858 |  | 51.066 | 7.146 | 48.957 - 53.2974 |  | 51.0601 | 7.146 | 48.952 - 53.2914 |  | 51.0597 | 7.146 | 48.951 - 53.2909 |  | 51.0788 | 7.147 | 48.971 - 53.3132 |  | 51.0273 | 7.143 | 48.921 - 53.2573 |  | 51.0409 | 7.144 | 48.934 - 53.2716 |  | 51.042 | 7.144 | 48.88 - 53.238 |  | 51.0441 | 7.145 | 48.942 - 53.1705 |  | 51.0437 | 7.144 | 48.722 - 53.2265 |  |
| τ00:id (between-subject) | 40.6148 | 6.373 | 27.594 - 62.2289 |  | 52.4003 | 7.239 | 35.839 - 79.8791 |  | 52.7664 | 7.264 | 36.088 - 80.4133 |  | 51.3715 | 7.167 | 35.099 - 78.3777 |  | 52.4809 | 7.244 | 35.848 - 80.009 |  | 50.4122 | 7.1 | 34.346 - 76.6323 |  | 54.1367 | 7.358 | 36.981 - 82.5346 |  | 52.9988 | 7.28 | 36.233 - 80.7746 |  | 53.1614 | 7.291 | 31.21 - 77.2988 |  | 52.4874 | 7.245 | 31.626 - 73.5629 |  | 52.8641 | 7.271 | 31.653 - 72.0282 |  |
| τ11: TIME (between-subject) | 5.46E-01 | 0.739 | 0.305 - 0.9704 |  | 5.24E-01 | 0.724 | 0.288 - 0.9453 |  | 5.06E-01 | 0.711 | 0.277 - 0.9157 |  | 5.53E-01 | 0.744 | 0.309 - 0.987 |  | 5.52E-01 | 0.743 | 0.309 - 0.9861 |  | 4.87E-01 | 0.698 | 0.257 - 0.8968 |  | 5.38E-01 | 0.733 | 0.302 - 0.9575 |  | 5.32E-01 | 0.729 | 0.294 - 0.9538 |  | 5.41E-01 | 0.736 | 0.239 - 0.8682 |  | 5.51E-01 | 0.742 | 0.246 - 0.8906 |  | 5.51E-01 | 0.742 | 0.25 - 0.8497 |  |
| **Model performance** |  |  |  |  |  |  |  |  |  |  |  |  |  |  |  |  |  |  |  |  |  |  |  |  |  |  |  |  |  |  |  |  |  |  |  |  |  |  |  |  |  |  |  |  |
| Deviance | 29773.6 |  |  |  | 29784.1 |  |  |  | 29786.3 |  |  |  | 29786.8 |  |  |  | 29787.8 |  |  |  | 29779.2 |  |  |  | 29786.2 |  |  |  | 29785.3 |  |  |  | 29786.3 |  |  |  | 29786.6 |  |  |  | 29786.9 |  |  |  |
| AIC | 29789.6 |  |  |  | 29800.1 |  |  |  | 29802.3 |  |  |  | 29802.8 |  |  |  | 29803.8 |  |  |  | 29795.2 |  |  |  | 29802.2 |  |  |  | 29801.3 |  |  |  | 29802.3 |  |  |  | 29802.6 |  |  |  | 29802.9 |  |  |  |
| log-Likelihood | -14887 |  |  |  | -14892 |  |  |  | -14893 |  |  |  | -14893 |  |  |  | -14894 |  |  |  | -14890 |  |  |  | -14893 |  |  |  | -14893 |  |  |  | -14893 |  |  |  | -14893 |  |  |  | -14893 |  |  |  |

**Conditional growth model – step time CV**

| **Predictors** | **Age** | | | | **Sex** | | | | **POMAB** | | | | **NPI** | | | | **Fall history** | | | | **No. of walks** | | | | **Falls during stay** | | | | **Hospitalization** | | | | **Antipsychotics** | | | | **Benzodiazepines** | | | | **Antidepressants** | | | |
| --- | --- | --- | --- | --- | --- | --- | --- | --- | --- | --- | --- | --- | --- | --- | --- | --- | --- | --- | --- | --- | --- | --- | --- | --- | --- | --- | --- | --- | --- | --- | --- | --- | --- | --- | --- | --- | --- | --- | --- | --- | --- | --- | --- | --- |
|  | **Estimate** | **SD** | **CI** | **p-value** | **Estimate** | **SD** | **CI** | **p-value** | **Estimate** | **SD** | **CI** | **p-value** | **Estimate** | **SD** | **CI** | **p-value** | **Estimate** | **SD** | **CI** | **p-value** | **Estimate** | **SD** | **CI** | **p-value** | **Estimate** | **SD** | **CI** | **p-value** | **Estimate** | **SD** | **CI** | **p-value** | **Estimate** | **SD** | **CI** | **p-value** | **Estimate** | **SD** | **CI** | **p-value** | **Estimate** | **SD** | **CI** | **p-value** |
| **Fixed effects** |  |  |  |  |  |  |  |  |  |  |  |  |  |  |  |  |  |  |  |  |  |  |  |  |  |  |  |  |  |  |  |  |  |  |  |  |  |  |  |  |  |  |  |  |
| Intercept) | 3.8176 | 10.0927 | -15.9637 – 23.5989 | 0.705 | 21.5413 | 1.4294 | 18.7397 – 24.3429 | **<0.001** | 27.2512 | 2.997 | 21.3771 – 33.1253 | **<0.001** | 18.8662 | 2.793 | 13.3919 – 24.3404 | **<0.001** | 19.3242 | 1.2367 | 16.9004 – 21.7481 | **<0.001** | 18.8364 | 1.6616 | 15.5796 – 22.0932 | **<0.001** | 19.3757 | 1.074 | 17.2707 – 21.4808 | **<0.001** | 19.4545 | 1.1736 | 17.1543 – 21.7547 | **<0.001** | 19.019 | 2.3553 | 14.4027 – 23.6353 | **<0.001** | 19.453 | 1.2534 | 16.9962 – 21.9097 | **<0.001** | 17.5367 | 1.4904 | 14.6155 – 20.4578 | **<0.001** |
| PREDICTOR | 0.2005 | 0.1319 | -0.0581 – 0.4591 | 0.129 | -4.558 | 1.9482 | -8.3764 – -0.7396 | **0.019** | -0.8425 | 0.2953 | -1.4212 – -0.2638 | **0.004** | 0.0054 | 0.0499 | -0.0924 – 0.1032 | 0.914 | -0.6622 | 2.2533 | -5.0785 – 3.7542 | 0.769 | 0.0006 | 0.0139 | -0.0266 – 0.0278 | 0.963 | -1.2101 | 1.2149 | -3.5913 – 1.1710 | 0.319 | 1.1622 | 0.3964 | 0.3853 – 1.9392 | **0.003** | 0.2596 | 0.7039 | -1.1201 – 1.6393 | 0.712 | 0.8043 | 0.3999 | 0.0204 – 1.5882 | **0.044** | 1.6316 | 0.4971 | 0.6573 – 2.6060 | **0.001** |
| TIME | -0.871 | 3.3548 | -7.4464 – 5.7044 | 0.795 | 0.9183 | 0.5069 | -0.0751 – 1.9118 | 0.07 | 0.291 | 1.0619 | -1.7903 – 2.3724 | 0.784 | -0.1822 | 0.9676 | -2.0785 – 1.7142 | 0.851 | 1.1994 | 0.4223 | 0.3716 – 2.0271 | **0.005** | 1.9404 | 0.557 | 0.8487 – 3.0321 | **<0.001** | 1.279 | 0.3623 | 0.5689 – 1.9892 | **<0.001** | -1.1953 | 2.4113 | -5.9215 – 3.5308 | 0.62 | 0.1563 | 2.6199 | -4.9787 – 5.2913 | 0.952 | -0.6679 | 2.1562 | -4.8940 – 3.5582 | 0.757 | 3.0171 | 2.0024 | -0.9075 – 6.9418 | 0.132 |
| PREDICTOR * TIME | 0.0273 | 0.0438 | -0.0586 – 0.1133 | 0.533 | 0.6078 | 0.6954 | -0.7552 – 1.9709 | 0.382 | 0.0953 | 0.1054 | -0.1113 – 0.3018 | 0.366 | 0.0266 | 0.0171 | -0.0070 – 0.0602 | 0.12 | 0.0753 | 0.7336 | -1.3625 – 1.5130 | 0.918 | -0.0071 | 0.0044 | -0.0156 – 0.0015 | 0.106 | 0.1179 | 0.1486 | -0.1734 – 0.4093 | 0.427 | 0.1846 | 0.762 | -1.3090 – 1.6781 | 0.809 | 1.2091 | 0.7969 | -0.3528 – 2.7710 | 0.129 | 1.1289 | 0.672 | -0.1881 – 2.4459 | 0.093 | -0.831 | 0.686 | -2.1755 – 0.5135 | 0.226 |
| **Random effects** |  |  |  |  |  |  |  |  |  |  |  |  |  |  |  |  |  |  |  |  |  |  |  |  |  |  |  |  |  |  |  |  |  |  |  |  |  |  |  |  |  |  |  |  |
| σ2 (within-subject) | 425.38 | 20.625 | 407.8 - 443.9838 |  | 425.301 | 20.623 | 407.728 - 443.8955 |  | 425.526 | 20.628 | 407.944 - 444.1324 |  | 425.211 | 20.621 | 407.648 - 443.7974 |  | 425.253 | 20.622 | 407.682 - 443.8461 |  | 425.37 | 20.624 | 407.796 - 443.9661 |  | 425.062 | 20.617 | 407.494 - 443.6516 |  | 425.342 | 20.624 | 407.768 - 443.9392 |  | 425.337 | 20.624 | 406.325 - 443.9256 |  | 425.591 | 20.63 | 408.221 - 443.1005 |  | 425.241 | 20.621 | 405.982 - 444.4901 |  |
| τ00:id (between-subject) | 33.1664 | 5.759 | 17.376 - 60.8943 |  | 29.8191 | 5.461 | 15.255 - 55.8936 |  | 27.4289 | 5.237 | 13.41 - 52.5369 |  | 35.5195 | 5.96 | 19.208 - 64.3409 |  | 36.1961 | 6.016 | 19.594 - 65.4732 |  | 35.2797 | 5.94 | 18.939 - 63.9906 |  | 36.7325 | 6.061 | 19.753 - 66.8503 |  | 35.4609 | 5.955 | 19.122 - 64.2711 |  | 36.1564 | 6.013 | 14.048 - 58.2131 |  | 35.1077 | 5.925 | 16.666 - 56.053 |  | 32.2464 | 5.679 | 11.585 - 50.9101 |  |
| τ11: TIME (between-subject) | ####### | 1.908 | 1.809 - 7.0509 |  | ####### | 1.951 | 1.925 - 7.2661 |  | ####### | 1.861 | 1.713 - 6.6892 |  | ####### | 1.885 | 1.799 - 6.7682 |  | ####### | 1.931 | 1.872 - 7.1411 |  | ####### | 1.837 | 1.658 - 6.5519 |  | ####### | 1.936 | 1.877 - 7.1989 |  | ####### | 1.879 | 1.727 - 6.8563 |  | ####### | 1.806 | 1.278 - 5.4069 |  | ####### | 1.739 | 1.141 - 4.7434 |  | ####### | 1.914 | 1.504 - 6.0472 |  |
| **Model performance** |  |  |  |  |  |  |  |  |  |  |  |  |  |  |  |  |  |  |  |  |  |  |  |  |  |  |  |  |  |  |  |  |  |  |  |  |  |  |  |  |  |  |  |  |
| Deviance | 38908.9 |  |  |  | 38906.8 |  |  |  | 38904.8 |  |  |  | 38909.5 |  |  |  | 38912 |  |  |  | 38909.6 |  |  |  | 38911.1 |  |  |  | 38911 |  |  |  | 38908.9 |  |  |  | 38908.7 |  |  |  | 38908.1 |  |  |  |
| AIC | 38924.9 |  |  |  | 38922.8 |  |  |  | 38920.8 |  |  |  | 38925.5 |  |  |  | 38928 |  |  |  | 38925.6 |  |  |  | 38927.1 |  |  |  | 38927 |  |  |  | 38924.9 |  |  |  | 38924.7 |  |  |  | 38924.1 |  |  |  |
| log-Likelihood | -19454 |  |  |  | -19453 |  |  |  | -19452 |  |  |  | -19455 |  |  |  | -19456 |  |  |  | -19455 |  |  |  | -19456 |  |  |  | -19455 |  |  |  | -19454 |  |  |  | -19454 |  |  |  | -19454 |  |  |  |

**Conditional growth model – step length CV**

| **Predictors** | **Age** | | | | **Sex** | | | | **POMAB** | | | | **NPI** | | | | **Fall history** | | | | **No. of walks** | | | | **Falls during stay** | | | | **Hospitalization** | | | | **Antipsychotics** | | | | **Benzodiazepines** | | | | **Antidepressants** | | | |
| --- | --- | --- | --- | --- | --- | --- | --- | --- | --- | --- | --- | --- | --- | --- | --- | --- | --- | --- | --- | --- | --- | --- | --- | --- | --- | --- | --- | --- | --- | --- | --- | --- | --- | --- | --- | --- | --- | --- | --- | --- | --- | --- | --- | --- |
|  | **Estimate** | **SD** | **CI** | **p-value** | **Estimate** | **SD** | **CI** | **p-value** | **Estimate** | **SD** | **CI** | **p-value** | **Estimate** | **SD** | **CI** | **p-value** | **Estimate** | **SD** | **CI** | **p-value** | **Estimate** | **SD** | **CI** | **p-value** | **Estimate** | **SD** | **CI** | **p-value** | **Estimate** | **SD** | **CI** | **p-value** | **Estimate** | **SD** | **CI** | **p-value** | **Estimate** | **SD** | **CI** | **p-value** | **Estimate** | **SD** | **CI** | **p-value** |
| **Fixed effects** |  |  |  |  |  |  |  |  |  |  |  |  |  |  |  |  |  |  |  |  |  |  |  |  |  |  |  |  |  |  |  |  |  |  |  |  |  |  |  |  |  |  |  |  |
| Intercept) | 4.198 | 12.7938 | -20.8773 – 29.2733 | 0.743 | 25.5268 | 1.8871 | 21.8281 – 29.2255 | **<0.001** | 34.2018 | 3.8177 | 26.7191 – 41.6844 | **<0.001** | 21.5969 | 3.5375 | 14.6635 – 28.5302 | **<0.001** | 22.6548 | 1.5556 | 19.6059 – 25.7036 | **<0.001** | 23.6552 | 2.0879 | 19.5629 – 27.7475 | **<0.001** | 23.8785 | 1.3657 | 21.2017 – 26.5553 | **<0.001** | 23.0367 | 1.5027 | 20.0914 – 25.9819 | **<0.001** | 20.7518 | 2.9667 | 14.9371 – 26.5665 | **<0.001** | 23.1279 | 1.6092 | 19.9739 – 26.2819 | **<0.001** | 23.5437 | 1.9853 | 19.6526 – 27.4347 | **<0.001** |
| PREDICTOR | 0.2514 | 0.167 | -0.0760 – 0.5788 | 0.132 | -3.9941 | 2.5654 | -9.0221 – 1.0339 | 0.119 | -1.1338 | 0.3777 | -1.8741 – -0.3935 | **0.003** | 0.0349 | 0.0631 | -0.0888 – 0.1586 | 0.58 | 2.422 | 2.8423 | -3.1487 – 7.9927 | 0.394 | -0.0051 | 0.0181 | -0.0406 – 0.0303 | 0.776 | -1.7127 | 1.3531 | -4.3647 – 0.9392 | 0.206 | 1.3583 | 0.4312 | 0.5131 – 2.2034 | **0.002** | 0.9274 | 0.7817 | -0.6047 – 2.4595 | 0.235 | 0.9894 | 0.4533 | 0.1008 – 1.8779 | **0.029** | 1.4402 | 0.5378 | 0.3862 – 2.4943 | **0.007** |
| TIME | 1.3192 | 3.6338 | -5.8030 – 8.4414 | 0.717 | 0.9552 | 0.5373 | -0.0978 – 2.0083 | 0.075 | 0.1907 | 1.1557 | -2.0744 – 2.4558 | 0.869 | 0.1556 | 1.0419 | -1.8865 – 2.1977 | 0.881 | 1.3978 | 0.4492 | 0.5175 – 2.2782 | **0.002** | 1.9122 | 0.5964 | 0.7432 – 3.0812 | **0.001** | 1.2285 | 0.3882 | 0.4676 – 1.9893 | **0.002** | 1.5245 | 3.0477 | -4.4489 – 7.4980 | 0.617 | 3.2497 | 3.3012 | -3.2206 – 9.7199 | 0.325 | 0.9102 | 2.7793 | -4.5371 – 6.3575 | 0.743 | -0.1928 | 2.6468 | -5.3804 – 4.9947 | 0.942 |
| PREDICTOR * TIME | -0.0008 | 0.0475 | -0.0939 – 0.0922 | 0.986 | 0.5903 | 0.7386 | -0.8574 – 2.0380 | 0.424 | 0.1105 | 0.1147 | -0.1144 – 0.3354 | 0.335 | 0.0209 | 0.0184 | -0.0152 – 0.0570 | 0.257 | -0.4156 | 0.7816 | -1.9475 – 1.1162 | 0.595 | -0.0065 | 0.0047 | -0.0157 – 0.0027 | 0.166 | 0.2593 | 0.1686 | -0.0712 – 0.5898 | 0.124 | -0.3962 | 0.8288 | -2.0206 – 1.2283 | 0.633 | 0.4217 | 0.8856 | -1.3141 – 2.1574 | 0.634 | 0.7511 | 0.7666 | -0.7513 – 2.2535 | 0.327 | -0.3541 | 0.7403 | -1.8052 – 1.0969 | 0.632 |
| **Random effects** |  |  |  |  |  |  |  |  |  |  |  |  |  |  |  |  |  |  |  |  |  |  |  |  |  |  |  |  |  |  |  |  |  |  |  |  |  |  |  |  |  |  |  |  |
| σ2 (within-subject) | 467.944 | 21.632 | 448.594 - 488.4244 |  | 468.005 | 21.633 | 448.653 - 488.4867 |  | 468.172 | 21.637 | 448.801 - 488.6733 |  | 467.899 | 21.631 | 448.552 - 488.3741 |  | 467.994 | 21.633 | 448.64 - 488.4781 |  | 468.065 | 21.635 | 448.704 - 488.5507 |  | 467.503 | 21.622 | 448.167 - 487.9668 |  | 468.03 | 21.634 | 448.676 - 488.5133 |  | 468.038 | 21.634 | 446.497 - 489.5824 |  | 468.042 | 21.634 | 446.416 - 487.429 | | 467.985 | 21.633 | 449.245 - 487.9353 |  |
| τ00:id (between-subject) | 63.7705 | 7.986 | 35.684 - 112.5942 |  | 63.0805 | 7.942 | 35.489 - 111.1501 |  | 52.2992 | 7.232 | 27.466 - 95.657 |  | 66.6242 | 8.162 | 37.918 - 116.6014 |  | 66.2853 | 8.142 | 37.6 - 115.6995 |  | 66.8217 | 8.174 | 37.885 - 116.8929 |  | 69.8805 | 8.359 | 39.724 - 121.3017 |  | 67.0382 | 8.188 | 37.98 - 117.5308 |  | 66.1297 | 8.132 | 35.218 - 98.0744 |  | 67.6515 | 8.225 | 33.674 - 100.8618 | | 67.6723 | 8.226 | 34.575 - 101.2944 |  |
| τ11: TIME (between-subject) | ####### | 2.081 | 2.243 - 8.0832 |  | ####### | 2.058 | 2.196 - 7.9007 |  | ####### | 2.008 | 2.036 - 7.6334 |  | ####### | 2.04 | 2.159 - 7.7765 |  | ####### | 2.048 | 2.164 - 7.8793 |  | ####### | 1.976 | 1.958 - 7.4504 |  | ####### | 2.076 | 2.24 - 8.0241 |  | ####### | 2.052 | 2.181 - 7.8834 |  | ####### | 2.046 | 1.599 - 6.5102 |  | 4.1267 | 2.031 | 1.564 - 6.5485 | | ####### | 2.064 | 1.814 - 6.8426 |  |
| **Model performance** |  |  |  |  |  |  |  |  |  |  |  |  |  |  |  |  |  |  |  |  |  |  |  |  |  |  |  |  |  |  |  |  |  |  |  |  |  |  |  |  |  |  |  |  |
| Deviance | 39341.6 |  |  |  | 39341.8 |  |  |  | 39336.1 |  |  |  | 39341.8 |  |  |  | 39343.5 |  |  |  | 39341.8 |  |  |  | 39341.9 |  |  |  | 39344.3 |  |  |  | 39342.9 |  |  |  | 39343.1 |  |  |  | 39344.3 |  |  |  |
| AIC | 39357.6 |  |  |  | 39357.8 |  |  |  | 39352.1 |  |  |  | 39357.8 |  |  |  | 39359.5 |  |  |  | 39357.8 |  |  |  | 39357.9 |  |  |  | 39360.3 |  |  |  | 39358.9 |  |  |  | 39359.1 |  |  |  | 39360.3 |  |  |  |
| log-Likelihood | -19671 |  |  |  | -19671 |  |  |  | -19668 |  |  |  | -19671 |  |  |  | -19672 |  |  |  | -19671 |  |  |  | -19671 |  |  |  | -19672 |  |  |  | -19671 |  |  |  | -19672 |  |  |  | -19672 |  |  |  |

**Conditional growth model – sacrum ML velocity**

| **Predictors** | **Age** | | | | **Sex** | | | | **POMAB** | | | | **NPI** | | | | **Fall history** | | | | **No. of walks** | | | | **Falls during stay** | | | | **Hospitalization** | | | | **Antipsychotics** | | | | **Benzodiazepines** | | | | **Antidepressants** | | | |
| --- | --- | --- | --- | --- | --- | --- | --- | --- | --- | --- | --- | --- | --- | --- | --- | --- | --- | --- | --- | --- | --- | --- | --- | --- | --- | --- | --- | --- | --- | --- | --- | --- | --- | --- | --- | --- | --- | --- | --- | --- | --- | --- | --- | --- |
|  | **Estimate** | **SD** | **CI** | **p-value** | **Estimate** | **SD** | **CI** | **p-value** | **Estimate** | **SD** | **CI** | **p-value** | **Estimate** | **SD** | **CI** | **p-value** | **Estimate** | **SD** | **CI** | **p-value** | **Estimate** | **SD** | **CI** | **p-value** | **Estimate** | **SD** | **CI** | **p-value** | **Estimate** | **SD** | **CI** | **p-value** | **Estimate** | **SD** | **CI** | **p-value** | **Estimate** | **SD** | **CI** | **p-value** | **Estimate** | **SD** | **CI** | **p-value** |
| **Fixed effects** |  |  |  |  |  |  |  |  |  |  |  |  |  |  |  |  |  |  |  |  |  |  |  |  |  |  |  |  |  |  |  |  |  |  |  |  |  |  |  |  |  |  |  |  |
| Intercept) | 23.912 | 3.5183 | 17.0162 – 30.8078 | **<0.001** | 18.289 | 0.477 | 17.3541 – 19.2238 | **<0.001** | 19.4059 | 1.1146 | 17.2214 – 21.5904 | **<0.001** | 16.1678 | 0.9612 | 14.2839 – 18.0516 | **<0.001** | 17.1145 | 0.4346 | 16.2628 – 17.9662 | **<0.001** | 17.4658 | 0.5936 | 16.3023 – 18.6292 | **<0.001** | 17.5614 | 0.3675 | 16.8411 – 18.2816 | **<0.001** | 17.2873 | 0.415 | 16.4739 – 18.1007 | **<0.001** | 17.9978 | 0.8269 | 16.3771 – 19.6185 | **<0.001** | 17.2039 | 0.4383 | 16.3447 – 18.0630 | **<0.001** | 16.9215 | 0.5308 | 15.8812 – 17.9617 | **<0.001** |
| PREDICTOR | -0.0857 | 0.046 | -0.1759 – 0.0044 | 0.062 | -1.6909 | 0.6506 | -2.9662 – -0.4157 | **0.009** | -0.2081 | 0.1097 | -0.4231 – 0.0070 | 0.058 | 0.0236 | 0.0172 | -0.0100 – 0.0573 | 0.169 | 0.8335 | 0.7918 | -0.7185 – 2.3855 | 0.293 | -0.0014 | 0.0049 | -0.0111 – 0.0082 | 0.77 | -0.9787 | 0.409 | -1.7803 – -0.1772 | **0.017** | 0.2014 | 0.1178 | -0.0295 – 0.4324 | 0.087 | -0.0002 | 0.2085 | -0.4088 – 0.4085 | 0.999 | 0.0838 | 0.1211 | -0.1535 – 0.3211 | 0.489 | 0.3122 | 0.1391 | 0.0396 – 0.5848 | **0.025** |
| TIME | 0.0047 | 0.9723 | -1.9010 – 1.9103 | 0.996 | 0.0836 | 0.1417 | -0.1941 – 0.3613 | 0.555 | -0.2899 | 0.3062 | -0.8900 – 0.3102 | 0.344 | -0.0911 | 0.2894 | -0.6583 – 0.4761 | 0.753 | 0.2542 | 0.1229 | 0.0133 – 0.4950 | **0.039** | 0.3137 | 0.1675 | -0.0145 – 0.6420 | 0.061 | 0.2555 | 0.1029 | 0.0537 – 0.4573 | **0.013** | 0.3897 | 0.8572 | -1.2904 – 2.0698 | 0.649 | -0.773 | 0.9196 | -2.5755 – 1.0294 | 0.401 | 0.5736 | 0.754 | -0.9042 – 2.0514 | 0.447 | 0.8551 | 0.7136 | -0.5435 – 2.2537 | 0.231 |
| PREDICTOR * TIME | 0.0025 | 0.0128 | -0.0224 – 0.0275 | 0.842 | 0.2143 | 0.1947 | -0.1674 – 0.5959 | 0.271 | 0.0495 | 0.03 | -0.0093 – 0.1083 | 0.099 | 0.0053 | 0.0051 | -0.0047 – 0.0153 | 0.296 | -0.1687 | 0.2112 | -0.5826 – 0.2452 | 0.424 | -0.0011 | 0.0012 | -0.0035 – 0.0013 | 0.362 | 0.0779 | 0.0519 | -0.0238 – 0.1796 | 0.133 | -0.0267 | 0.2249 | -0.4676 – 0.4141 | 0.905 | 0.2529 | 0.2368 | -0.2113 – 0.7170 | 0.286 | 0.3043 | 0.199 | -0.0857 – 0.6943 | 0.126 | -0.2316 | 0.1958 | -0.6154 – 0.1521 | 0.237 |
| **Random effects** |  |  |  |  |  |  |  |  |  |  |  |  |  |  |  |  |  |  |  |  |  |  |  |  |  |  |  |  |  |  |  |  |  |  |  |  |  |  |  |  |  |  |  |  |
| σ2 (within-subject) | 59.3859 | 7.706 | 56.932 - 61.9821 |  | 59.4409 | 7.71 | 56.983 - 62.0424 |  | 59.3857 | 7.706 | 56.931 - 61.9835 |  | 59.3674 | 7.705 | 56.915 - 61.963 |  | 59.2426 | 7.697 | 56.787 - 61.842 |  | 59.3786 | 7.706 | 56.925 - 61.9747 |  | 59.3149 | 7.702 | 56.865 - 61.9078 |  | 59.3499 | 7.704 | 56.854 - 61.6133 |  | 59.3443 | 7.704 | 56.899 - 61.7711 |  | 59.3741 | 7.705 | 56.848 - 61.8541 | | 59.3662 | 7.705 | 56.681 - 61.813 |  |
| τ00:id (between-subject) | 3.739 | 1.934 | 1.676 - 7.4688 |  | 2.9276 | 1.711 | 0.985 - 6.4776 |  | 3.7848 | 1.945 | 1.628 - 7.6465 |  | 3.8618 | 1.965 | 1.726 - 7.697 |  | 4.2252 | 2.056 | 1.932 - 8.2956 |  | 4.2855 | 2.07 | 1.987 - 8.374 |  | 3.9584 | 1.99 | 1.819 - 7.7952 |  | 4.2306 | 2.057 | 1.669 - 6.8501 |  | 4.1941 | 2.048 | 1.696 - 6.7472 |  | 4.0085 | 2.002 | 1.482 - 6.1441 | | 3.9288 | 1.982 | 1.443 - 6.5969 |  |
| τ11: TIME (between-subject) | 2.18E-01 | 0.467 | 0.083 - 0.4821 |  | 2.05E-01 | 0.453 | 0.075 - 0.4594 |  | 1.95E-01 | 0.442 | 0.066 - 0.4499 |  | 2.14E-01 | 0.463 | 0.084 - 0.4738 |  | 2.23E-01 | 0.472 | 0.083 - 0.4944 |  | 2.10E-01 | 0.458 | 0.076 - 0.4731 |  | 1.96E-01 | 0.443 | 0.07 - 0.4434 |  | 2.26E-01 | 0.475 | 0.046 - 0.3949 |  | 2.15E-01 | 0.463 | 0.026 - 0.3921 |  | 0.1924 | 0.439 | 0.04 - 0.3412 | | 2.07E-01 | 0.455 | 0.051 - 0.3728 |  |
| **Model performance** |  |  |  |  |  |  |  |  |  |  |  |  |  |  |  |  |  |  |  |  |  |  |  |  |  |  |  |  |  |  |  |  |  |  |  |  |  |  |  |  |  |  |  |  |
| Deviance | 30293 |  |  |  | 30290.8 |  |  |  | 30292.1 |  |  |  | 30291.8 |  |  |  | 30099.7 |  |  |  | 30295.5 |  |  |  | 30290.3 |  |  |  | 30294.9 |  |  |  | 30293.8 |  |  |  | 30290.9 |  |  |  | 30293.1 |  |  |  |
| AIC | 30309 |  |  |  | 30306.8 |  |  |  | 30308.1 |  |  |  | 30307.8 |  |  |  | 30115.7 |  |  |  | 30311.5 |  |  |  | 30306.3 |  |  |  | 30310.9 |  |  |  | 30309.8 |  |  |  | 30306.9 |  |  |  | 30309.1 |  |  |  |
| log-Likelihood | -15146 |  |  |  | -15145 |  |  |  | -15146 |  |  |  | -15146 |  |  |  | -15050 |  |  |  | -15148 |  |  |  | -15145 |  |  |  | -15147 |  |  |  | -15147 |  |  |  | -15145 |  |  |  | -15147 |  |  |  |

**Conditional growth model – walking speed**

| **Predictors** | **Age** | | | | **Sex** | | | | **POMAB** | | | | **NPI** | | | | **Fall history** | | | | **No. of walks** | | | | **Falls during stay** | | | | **Hospitalization** | | | | **Antipsychotics** | | | | **Benzodiazepines** | | | | **Antidepressants** | | | |
| --- | --- | --- | --- | --- | --- | --- | --- | --- | --- | --- | --- | --- | --- | --- | --- | --- | --- | --- | --- | --- | --- | --- | --- | --- | --- | --- | --- | --- | --- | --- | --- | --- | --- | --- | --- | --- | --- | --- | --- | --- | --- | --- | --- | --- |
|  | **Estimate** | **SD** | **CI** | **p-value** | **Estimate** | **SD** | **CI** | **p-value** | **Estimate** | **SD** | **CI** | **p-value** | **Estimate** | **SD** | **CI** | **p-value** | **Estimate** | **SD** | **CI** | **p-value** | **Estimate** | **SD** | **CI** | **p-value** | **Estimate** | **SD** | **CI** | **p-value** | **Estimate** | **SD** | **CI** | **p-value** | **Estimate** | **SD** | **CI** | **p-value** | **Estimate** | **SD** | **CI** | **p-value** | **Estimate** | **SD** | **CI** | **p-value** |
| **Fixed effects** |  |  |  |  |  |  |  |  |  |  |  |  |  |  |  |  |  |  |  |  |  |  |  |  |  |  |  |  |  |  |  |  |  |  |  |  |  |  |  |  |  |  |  |  |
| Intercept) | 101.0647 | 16.1878 | 69.3371 – 132.7922 | **<0.001** | 53.9521 | 2.5972 | 48.8618 – 59.0425 | **<0.001** | 52.8102 | 5.7044 | 41.6299 – 63.9906 | **<0.001** | 45.0974 | 4.7603 | 35.7674 – 54.4273 | **<0.001** | 51.8814 | 2.1054 | 47.7548 – 56.0080 | **<0.001** | 47.6852 | 2.7276 | 42.3393 – 53.0311 | **<0.001** | 50.2726 | 1.8092 | 46.7266 – 53.8185 | **<0.001** | 50.6434 | 2.0723 | 46.5817 – 54.7050 | **<0.001** | 53.4613 | 4.1061 | 45.4134 – 61.5092 | **<0.001** | 49.9227 | 2.182 | 45.6461 – 54.1993 | **<0.001** | 50.6489 | 2.7267 | 45.3046 – 55.9932 | **<0.001** |
| PREDICTOR | -0.6601 | 0.2109 | -1.0733 – -0.2468 | **0.002** | -6.0051 | 3.496 | -12.8572 – 0.8470 | 0.086 | -0.2347 | 0.572 | -1.3559 – 0.8864 | 0.682 | 0.1058 | 0.0847 | -0.0602 – 0.2718 | 0.212 | -4.2796 | 3.8633 | -11.8515 – 3.2923 | 0.268 | 0.0372 | 0.0253 | -0.0124 – 0.0869 | 0.142 | 1.4568 | 0.9363 | -0.3783 – 3.2919 | 0.12 | -0.3291 | 0.2804 | -0.8786 – 0.2204 | 0.241 | -0.8791 | 0.5099 | -1.8786 – 0.1203 | 0.085 | -0.2511 | 0.2952 | -0.8298 – 0.3276 | 0.395 | -0.5332 | 0.3523 | -1.2236 – 0.1572 | 0.13 |
| TIME | -2.2588 | 2.3357 | -6.8368 – 2.3191 | 0.334 | -0.2394 | 0.3507 | -0.9267 – 0.4479 | 0.495 | 0.1057 | 0.763 | -1.3899 – 1.6012 | 0.89 | -0.3564 | 0.6745 | -1.6785 – 0.9656 | 0.597 | -0.4183 | 0.2923 | -0.9912 – 0.1546 | 0.152 | -0.7374 | 0.3915 | -1.5047 – 0.0298 | 0.06 | -0.483 | 0.2485 | -0.9699 – 0.0040 | 0.052 | -0.1798 | 4.0931 | -8.2021 – 7.8425 | 0.965 | -3.5398 | 4.5541 | -12.4657 – 5.3861 | 0.437 | 1.9996 | 3.7775 | -5.4041 – 9.4033 | 0.597 | -0.1099 | 3.6041 | -7.1738 – 6.9540 | 0.976 |
| PREDICTOR * TIME | 0.0239 | 0.0305 | -0.0358 – 0.0837 | 0.433 | -0.3616 | 0.4809 | -1.3041 – 0.5809 | 0.452 | -0.056 | 0.076 | -0.2050 – 0.0929 | 0.461 | -0.0014 | 0.012 | -0.0249 – 0.0220 | 0.904 | -0.0121 | 0.5138 | -1.0191 – 0.9950 | 0.981 | 0.0029 | 0.0032 | -0.0034 – 0.0092 | 0.363 | -0.1463 | 0.1112 | -0.3643 – 0.0717 | 0.188 | -0.335 | 0.5421 | -1.3976 – 0.7276 | 0.537 | 0.5804 | 0.5762 | -0.5490 – 1.7098 | 0.314 | -0.4967 | 0.5039 | -1.4843 – 0.4909 | 0.324 | 0.2081 | 0.4808 | -0.7342 – 1.1504 | 0.66 |
| **Random effects** |  |  |  |  |  |  |  |  |  |  |  |  |  |  |  |  |  |  |  |  |  |  |  |  |  |  |  |  |  |  |  |  |  |  |  |  |  |  |  |  |  |  |  |  |
| σ2 (within-subject) | 149.039 | 12.208 | 142.884 - 155.5541 |  | 149.019 | 12.207 | 142.865 - 155.5321 |  | 149.041 | 12.208 | 142.885 - 155.5547 |  | 149.03 | 12.208 | 142.874 - 155.5434 |  | 149.037 | 12.208 | 142.882 - 155.5515 |  | 149.03 | 12.208 | 142.876 - 155.5442 |  | 148.951 | 12.205 | 142.799 - 155.4609 |  | 148.846 | 12.2 | 142.698 - 155.3523 |  | 148.842 | 12.2 | 34455.3 |  | 148.831 | 12.2 | 142.855 - 155.6323 | | 148.839 | 12.2 | 142.728 - 155.0212 |  |
| τ00:id (between-subject) | 136.511 | 11.684 | 93.149 - 208.5707 |  | 154.266 | 12.42 | 105.689 - 234.8713 |  | 162.465 | 12.746 | 111.418 - 247.2402 |  | 158.181 | 12.577 | 108.469 - 240.7239 |  | 159.323 | 12.622 | 109.151 - 242.6169 |  | 156.93 | 12.527 | 107.604 - 238.7002 |  | 164.92 | 12.842 | 113.103 - 250.9391 |  | 163.513 | 12.787 | 112.129 - 248.7473 |  | 161.368 | 12.703 | 34471.3 |  | 162.374 | 12.743 | 97.905 - 228.382 | | 162.707 | 12.756 | 99.799 - 216.9534 |  |
| τ11: TIME (between-subject) | ####### | 1.411 | 1.14 - 3.4702 |  | ####### | 1.427 | 1.158 - 3.5892 |  | ####### | 1.411 | 1.136 - 3.4981 |  | ####### | 1.427 | 1.162 - 3.5638 |  | ####### | 1.427 | 1.165 - 3.5603 |  | ####### | 1.42 | 1.147 - 3.5385 |  | ####### | 1.417 | 1.15 - 3.517 |  | ####### | 1.425 | 1.157 - 3.5607 |  | ####### | 1.403 | -17228 |  | 2.0115 | 1.418 | 0.947 - 3.173 | | ####### | 1.421 | 0.909 - 3.0769 |  |
| **Model performance** |  |  |  |  |  |  |  |  |  |  |  |  |  |  |  |  |  |  |  |  |  |  |  |  |  |  |  |  |  |  |  |  |  |  |  |  |  |  |  |  |  |  |  |  |
| Deviance | 34452.7 |  |  |  | 34456.3 |  |  |  | 34460.9 |  |  |  | 34460.4 |  |  |  | 34460.6 |  |  |  | 34456.8 |  |  |  | 34459.7 |  |  |  | 34456 |  |  |  | 34455.3 |  |  |  | 34455.5 |  |  |  | 34456.2 |  |  |  |
| AIC | 34468.7 |  |  |  | 34472.3 |  |  |  | 34476.9 |  |  |  | 34476.4 |  |  |  | 34476.6 |  |  |  | 34472.8 |  |  |  | 34475.7 |  |  |  | 34472 |  |  |  | 34471.3 |  |  |  | 34471.5 |  |  |  | 34472.2 |  |  |  |
| log-Likelihood | -17226 |  |  |  | -17228 |  |  |  | -17230 |  |  |  | -17230 |  |  |  | -17230 |  |  |  | -17228 |  |  |  | -17230 |  |  |  | -17228 |  |  |  | -17228 |  |  |  | -17228 |  |  |  | -17228 |  |  |  |

**Conditional growth model – step width**

| **Predictors** | **Age** | | | | **Sex** | | | | **POMAB** | | | | **NPI** | | | | **Fall history** | | | | **No. of walks** | | | | **Falls during stay** | | | | **Hospitalization** | | | | **Antipsychotics** | | | | **Benzodiazepines** | | | | **Antidepressants** | | | |
| --- | --- | --- | --- | --- | --- | --- | --- | --- | --- | --- | --- | --- | --- | --- | --- | --- | --- | --- | --- | --- | --- | --- | --- | --- | --- | --- | --- | --- | --- | --- | --- | --- | --- | --- | --- | --- | --- | --- | --- | --- | --- | --- | --- | --- |
|  | **Estimate** | **SD** | **CI** | **p-value** | **Estimate** | **SD** | **CI** | **p-value** | **Estimate** | **SD** | **CI** | **p-value** | **Estimate** | **SD** | **CI** | **p-value** | **Estimate** | **SD** | **CI** | **p-value** | **Estimate** | **SD** | **CI** | **p-value** | **Estimate** | **SD** | **CI** | **p-value** | **Estimate** | **SD** | **CI** | **p-value** | **Estimate** | **SD** | **CI** | **p-value** | **Estimate** | **SD** | **CI** | **p-value** | **Estimate** | **SD** | **CI** | **p-value** |
| **Fixed effects** |  |  |  |  |  |  |  |  |  |  |  |  |  |  |  |  |  |  |  |  |  |  |  |  |  |  |  |  |  |  |  |  |  |  |  |  |  |  |  |  |  |  |  |  |
| Intercept) | 24.5075 | 4.6443 | 15.4049 – 33.6101 | **<0.001** | 15.1256 | 0.6686 | 13.8152 – 16.4360 | **<0.001** | 13.4423 | 1.481 | 10.5396 – 16.3450 | **<0.001** | 18.1288 | 1.2852 | 15.6099 – 20.6477 | **<0.001** | 16.3022 | 0.5716 | 15.1818 – 17.4225 | **<0.001** | 17.0906 | 0.7474 | 15.6256 – 18.5555 | **<0.001** | 16.6648 | 0.4844 | 15.7154 – 17.6142 | **<0.001** | 16.1455 | 0.5462 | 15.0750 – 17.2160 | **<0.001** | 16.4975 | 1.1164 | 14.3094 – 18.6856 | **<0.001** | 16.2766 | 0.5864 | 15.1272 – 17.4259 | **<0.001** | 16.1757 | 0.7359 | 14.7334 – 17.6180 | **<0.001** |
| PREDICTOR | -0.1035 | 0.0605 | -0.2221 – 0.0150 | 0.087 | 2.6674 | 0.9005 | 0.9025 – 4.4323 | **0.003** | 0.334 | 0.1484 | 0.0430 – 0.6249 | **0.024** | -0.0292 | 0.0229 | -0.0741 – 0.0156 | 0.201 | 1.0145 | 1.0487 | -1.0410 – 3.0700 | 0.333 | -0.006 | 0.0069 | -0.0196 – 0.0076 | 0.385 | -0.3151 | 0.2485 | -0.8022 – 0.1720 | 0.205 | 0.0599 | 0.0723 | -0.0818 – 0.2016 | 0.407 | 0.0143 | 0.1342 | -0.2487 – 0.2773 | 0.915 | 0.0423 | 0.0767 | -0.1080 – 0.1927 | 0.581 | 0.118 | 0.0911 | -0.0606 – 0.2965 | 0.195 |
| TIME | -0.4147 | 0.6075 | -1.6054 – 0.7761 | 0.495 | 0.1527 | 0.0895 | -0.0227 – 0.3281 | 0.088 | 0.1895 | 0.1998 | -0.2022 – 0.5811 | 0.343 | -0.2548 | 0.171 | -0.5901 – 0.0804 | 0.136 | 0.0447 | 0.0759 | -0.1040 – 0.1934 | 0.556 | 0.0423 | 0.1021 | -0.1578 – 0.2424 | 0.679 | 0.0833 | 0.0655 | -0.0450 – 0.2117 | 0.203 | 1.7741 | 1.0797 | -0.3420 – 3.8902 | 0.1 | 0.1224 | 1.2382 | -2.3044 – 2.5493 | 0.921 | 0.9826 | 1.0152 | -1.0071 – 2.9723 | 0.333 | 0.7488 | 0.9727 | -1.1576 – 2.6552 | 0.441 |
| PREDICTOR * TIME | 0.0062 | 0.0079 | -0.0093 – 0.0218 | 0.431 | -0.1632 | 0.1227 | -0.4037 – 0.0773 | 0.184 | -0.0134 | 0.0198 | -0.0523 – 0.0254 | 0.499 | 0.006 | 0.003 | 0.0001 – 0.0119 | 0.0516 | 0.043 | 0.1337 | -0.2191 – 0.3051 | 0.748 | 0.0003 | 0.0008 | -0.0014 – 0.0019 | 0.748 | 0.0219 | 0.0306 | -0.0381 – 0.0820 | 0.474 | -0.0053 | 0.1395 | -0.2786 – 0.2680 | 0.97 | 0.0619 | 0.1514 | -0.2349 – 0.3586 | 0.683 | 0.0506 | 0.1299 | -0.2040 – 0.3051 | 0.697 | -0.1034 | 0.1245 | -0.3474 – 0.1406 | 0.406 |
| **Random effects** |  |  |  |  |  |  |  |  |  |  |  |  |  |  |  |  |  |  |  |  |  |  |  |  |  |  |  |  |  |  |  |  |  |  |  |  |  |  |  |  |  |  |  |  |
| σ2 (within-subject) | 12.0656 | 3.474 | 11.567 - 12.5934 |  | 12.0658 | 3.474 | 11.567 - 12.5936 |  | 12.0655 | 3.474 | 11.567 - 12.5933 |  | 12.0613 | 3.473 | 11.563 - 12.5887 |  | 12.0644 | 3.473 | 11.566 - 12.592 |  | 12.0658 | 3.474 | 11.567 - 12.5936 |  | 12.0623 | 3.473 | 11.564 - 12.5902 |  | 12.0795 | 3.476 | 11.58 - 12.6079 |  | 12.0787 | 3.475 | 11.592 - 12.6089 |  | 12.0787 | 3.475 | 11.607 - 12.5726 | | 12.0784 | 3.475 | 11.515 - 12.5746 |  |
| τ00:id (between-subject) | 11.2659 | 3.356 | 7.673 - 17.2309 |  | 10.1247 | 3.182 | 6.868 - 15.5175 |  | 10.8464 | 3.293 | 7.345 - 16.5809 |  | 11.4776 | 3.388 | 7.811 - 17.5116 |  | 11.6946 | 3.42 | 7.971 - 17.8699 |  | 11.7378 | 3.426 | 7.993 - 17.9282 |  | 11.7418 | 3.427 | 7.983 - 17.9649 |  | 11.2798 | 3.359 | 7.678 - 17.2971 |  | 11.8822 | 3.447 | 7.105 - 16.7507 |  | 11.6661 | 3.416 | 7.03 - 16.8051 | | 11.7939 | 3.434 | 7.333 - 16.2116 |  |
| τ11: TIME (between-subject) | 1.31E-01 | 0.362 | 0.069 - 0.2382 |  | 1.25E-01 | 0.354 | 0.066 - 0.2283 |  | 1.33E-01 | 0.365 | 0.069 - 0.2445 |  | 1.21E-01 | 0.348 | 0.063 - 0.2213 |  | 1.34E-01 | 0.366 | 0.071 - 0.2432 |  | 1.33E-01 | 0.364 | 0.069 - 0.2407 |  | 1.38E-01 | 0.371 | 0.071 - 0.2514 |  | 1.30E-01 | 0.361 | 0.068 - 0.2364 |  | 1.31E-01 | 0.362 | 0.058 - 0.2044 |  | 0.1305 | 0.361 | 0.055 - 0.2076 | | 1.31E-01 | 0.362 | 0.064 - 0.2015 |  |
| **Model performance** |  |  |  |  |  |  |  |  |  |  |  |  |  |  |  |  |  |  |  |  |  |  |  |  |  |  |  |  |  |  |  |  |  |  |  |  |  |  |  |  |  |  |  |  |
| Deviance | 23478 |  |  |  | 23472.6 |  |  |  | 23475.4 |  |  |  | 23477.1 |  |  |  | 23478.6 |  |  |  | 23480.1 |  |  |  | 23478.8 |  |  |  | 23481.2 |  |  |  | 23485.1 |  |  |  | 23483 |  |  |  | 23484.7 |  |  |  |
| AIC | 23494 |  |  |  | 23488.6 |  |  |  | 23491.4 |  |  |  | 23493.1 |  |  |  | 23494.6 |  |  |  | 23496.1 |  |  |  | 23494.8 |  |  |  | 23497.2 |  |  |  | 23501.1 |  |  |  | 23499 |  |  |  | 23500.7 |  |  |  |
| log-Likelihood | -11739 |  |  |  | -11736 |  |  |  | -11738 |  |  |  | -11739 |  |  |  | -11739 |  |  |  | -11740 |  |  |  | -11739 |  |  |  | -11741 |  |  |  | -11743 |  |  |  | -11741 |  |  |  | -11742 |  |  |  |

**Conditional growth model – step width** CV

| **Predictors** | **Age** | | | | **Sex** | | | | **POMAB** | | | | **NPI** | | | | **Fall history** | | | | **No. of walks** | | | | **Falls during stay** | | | | **Hospitalization** | | | | **Antipsychotics** | | | | **Benzodiazepines** | | | | **Antidepressants** | | | |
| --- | --- | --- | --- | --- | --- | --- | --- | --- | --- | --- | --- | --- | --- | --- | --- | --- | --- | --- | --- | --- | --- | --- | --- | --- | --- | --- | --- | --- | --- | --- | --- | --- | --- | --- | --- | --- | --- | --- | --- | --- | --- | --- | --- | --- |
|  | **Estimate** | **SD** | **CI** | **p-value** | **Estimate** | **SD** | **CI** | **p-value** | **Estimate** | **SD** | **CI** | **p-value** | **Estimate** | **SD** | **CI** | **p-value** | **Estimate** | **SD** | **CI** | **p-value** | **Estimate** | **SD** | **CI** | **p-value** | **Estimate** | **SD** | **CI** | **p-value** | **Estimate** | **SD** | **CI** | **p-value** | **Estimate** | **SD** | **CI** | **p-value** | **Estimate** | **SD** | **CI** | **p-value** | **Estimate** | **SD** | **CI** | **p-value** |
| **Fixed effects** |  |  |  |  |  |  |  |  |  |  |  |  |  |  |  |  |  |  |  |  |  |  |  |  |  |  |  |  |  |  |  |  |  |  |  |  |  |  |  |  |  |  |  |  |
| Intercept) | 32.1053 | 14.0295 | 4.6079 – 59.6027 | **0.022** | 37.0918 | 1.9215 | 33.3257 – 40.8578 | **<0.001** | 49.1988 | 3.6471 | 42.0505 – 56.3470 | **<0.001** | 30.5631 | 3.8028 | 23.1097 – 38.0165 | **<0.001** | 33.8225 | 1.6696 | 30.5502 – 37.0948 | **<0.001** | 31.6483 | 2.2255 | 27.2864 – 36.0102 | **<0.001** | 32.8002 | 1.4436 | 29.9709 – 35.6295 | **<0.001** | 33.3849 | 1.6287 | 30.1928 – 36.5771 | **<0.001** | 31.7877 | 3.2584 | 25.4012 – 38.1741 | **<0.001** | 33.7977 | 1.7232 | 30.4204 – 37.1751 | **<0.001** | 32.1028 | 2.14 | 27.9086 – 36.2971 | **<0.001** |
| PREDICTOR | 0.0101 | 0.1831 | -0.3487 – 0.3689 | 0.956 | -7.7254 | 2.6027 | -12.8265 – -2.6242 | **0.003** | -1.6854 | 0.3616 | -2.3942 – -0.9767 | **<0.001** | 0.0455 | 0.0678 | -0.0874 – 0.1784 | 0.502 | -3.1458 | 3.0523 | -9.1283 – 2.8367 | 0.303 | 0.0105 | 0.0196 | -0.0280 – 0.0490 | 0.591 | 0.0495 | 1.1757 | -2.2548 – 2.3538 | 0.966 | 0.0969 | 0.2363 | -0.3662 – 0.5601 | 0.682 | -0.3067 | 0.4317 | -1.1529 – 0.5394 | 0.477 | -0.3393 | 0.2499 | -0.8292 – 0.1506 | 0.175 | -0.0242 | 0.2812 | -0.5753 – 0.5270 | 0.932 |
| TIME | -0.7893 | 2.0166 | -4.7417 – 3.1631 | 0.695 | -0.1196 | 0.2966 | -0.7008 – 0.4617 | 0.687 | -1.5174 | 0.5372 | -2.5704 – -0.4645 | **0.005** | -0.4615 | 0.6257 | -1.6878 – 0.7648 | 0.461 | 0.0758 | 0.2498 | -0.4139 – 0.5655 | 0.762 | 0.4924 | 0.3487 | -0.1910 – 1.1757 | 0.158 | 0.0742 | 0.2215 | -0.3599 – 0.5083 | 0.738 | -1.8258 | 3.2844 | -8.2632 – 4.6116 | 0.578 | 1.3641 | 3.6173 | -5.7256 – 8.4538 | 0.706 | -2.2186 | 2.9647 | -8.0293 – 3.5920 | 0.454 | 1.3443 | 2.8476 | -4.2370 – 6.9256 | 0.637 |
| PREDICTOR * TIME | 0.0104 | 0.0266 | -0.0418 – 0.0626 | 0.695 | 0.1929 | 0.4038 | -0.5985 – 0.9844 | 0.633 | 0.1472 | 0.0504 | 0.0483 – 0.2460 | **0.004** | 0.0082 | 0.0109 | -0.0131 – 0.0295 | 0.45 | -0.2089 | 0.4259 | -1.0437 – 0.6259 | 0.624 | -0.0038 | 0.0022 | -0.0081 – 0.0005 | 0.084 | -0.0626 | 0.1463 | -0.3494 – 0.2243 | 0.669 | -0.3791 | 0.4598 | -1.2803 – 0.5220 | 0.41 | 0.3918 | 0.489 | -0.5666 – 1.3502 | 0.423 | 0.7404 | 0.3844 | -0.0131 – 1.4938 | 0.054 | 0.057 | 0.4093 | -0.7453 – 0.8593 | 0.88 |
| **Random effects** |  |  |  |  |  |  |  |  |  |  |  |  |  |  |  |  |  |  |  |  |  |  |  |  |  |  |  |  |  |  |  |  |  |  |  |  |  |  |  |  |  |  |  |  |
| σ2 (within-subject) | 464.776 | 21.559 | 445.592 - 485.0767 |  | 464.972 | 21.563 | 445.77 - 485.2943 |  | 465.49 | 21.575 | 446.36 - 485.7292 |  | 464.649 | 21.556 | 445.471 - 484.9401 |  | 464.754 | 21.558 | 445.576 - 485.0466 |  | 464.81 | 21.559 | 445.629 - 485.1074 |  | 464.692 | 21.557 | 445.505 - 484.9935 |  | 464.869 | 21.561 | 445.683 - 485.1761 |  | 464.825 | 21.56 | 443.916 - 485.6079 |  | 464.949 | 21.563 | 446.08 - 485.1973 | | 464.855 | 21.56 | 443.207 - 485.4445 |  |
| τ00:id (between-subject) | 85.4805 | 9.246 | 53.922 - 138.047 |  | 68.6666 | 8.287 | 41.915 - 113.8535 |  | 50.895 | 7.134 | 29.256 - 86.7364 |  | 83.8144 | 9.155 | 52.81 - 135.5279 |  | 83.2255 | 9.123 | 52.527 - 134.7714 |  | 82.9465 | 9.107 | 52.147 - 134.6832 |  | 84.7095 | 9.204 | 53.409 - 137.2943 |  | 85.6879 | 9.257 | 53.963 - 138.5308 |  | 85.7994 | 9.263 | 47.044 - 124.9463 |  | 84.2013 | 9.176 | 43.427 - 119.9995 | | 84.968 | 9.218 | 45.083 - 128.2571 |  |
| τ11: TIME (between-subject) | 4.34E-01 | 0.659 | 0 - 1.511 |  | 3.98E-01 | 0.631 | 0 - 1.466 |  | 1.70E-02 | 0.13 | 0 - 0.9083 |  | 4.39E-01 | 0.662 | 0 - 1.5213 |  | 4.15E-01 | 0.644 | 0 - 1.4659 |  | 2.77E-01 | 0.526 | 0 - 1.2438 |  | 3.96E-01 | 0.63 | 0 - 1.465 |  | 4.35E-01 | 0.659 | 0.001 - 1.5021 |  | 4.25E-01 | 0.652 | 0.002 - 1.2536 |  | 0.2637 | 0.514 | 0.001 - 0.8814 | | 4.34E-01 | 0.659 | 0.004 - 1.0816 |  |
| **Model performance** |  |  |  |  |  |  |  |  |  |  |  |  |  |  |  |  |  |  |  |  |  |  |  |  |  |  |  |  |  |  |  |  |  |  |  |  |  |  |  |  |  |  |  |  |
| Deviance | 39270.3 |  |  |  | 39261.3 |  |  |  | 39253.9 |  |  |  | 39268.6 |  |  |  | 39268.1 |  |  |  | 39267.7 |  |  |  | 39269.8 |  |  |  | 39269.2 |  |  |  | 39269.7 |  |  |  | 39267.7 |  |  |  | 39270.8 |  |  |  |
| AIC | 39286.3 |  |  |  | 39277.3 |  |  |  | 39269.9 |  |  |  | 39284.6 |  |  |  | 39284.1 |  |  |  | 39283.7 |  |  |  | 39285.8 |  |  |  | 39285.2 |  |  |  | 39285.7 |  |  |  | 39283.7 |  |  |  | 39286.8 |  |  |  |
| log-Likelihood | -19635 |  |  |  | -19631 |  |  |  | -19627 |  |  |  | -19634 |  |  |  | -19634 |  |  |  | -19634 |  |  |  | -19635 |  |  |  | -19635 |  |  |  | -19635 |  |  |  | -19634 |  |  |  | -19635 |  |  |  |

**Conditional growth model - Sacrum ML ROM**

| **Predictors** | **Age** | | | | **Sex** | | | | **POMAB** | | | | **NPI** | | | | **Fall history** | | | | **No. of walks** | | | | **Falls during stay** | | | | **Hospitalization** | | | | **Antipsychotics** | | | | **Benzodiazepines** | | | | **Antidepressants** | | | |
| --- | --- | --- | --- | --- | --- | --- | --- | --- | --- | --- | --- | --- | --- | --- | --- | --- | --- | --- | --- | --- | --- | --- | --- | --- | --- | --- | --- | --- | --- | --- | --- | --- | --- | --- | --- | --- | --- | --- | --- | --- | --- | --- | --- | --- |
|  | **Estimate** | **SD** | **CI** | **p-value** | **Estimate** | **SD** | **CI** | **p-value** | **Estimate** | **SD** | **CI** | **p-value** | **Estimate** | **SD** | **CI** | **p-value** | **Estimate** | **SD** | **CI** | **p-value** | **Estimate** | **SD** | **CI** | **p-value** | **Estimate** | **SD** | **CI** | **p-value** | **Estimate** | **SD** | **CI** | **p-value** | **Estimate** | **SD** | **CI** | **p-value** | **Estimate** | **SD** | **CI** | **p-value** | **Estimate** | **SD** | **CI** | **p-value** |
| **Fixed effects** |  |  |  |  |  |  |  |  |  |  |  |  |  |  |  |  |  |  |  |  |  |  |  |  |  |  |  |  |  |  |  |  |  |  |  |  |  |  |  |  |  |  |  |  |
| Intercept) | 19.3434 | 9.3801 | 0.9588 – 37.7281 | **0.039** | 24.4486 | 1.3106 | 21.8799 – 27.0173 | **<0.001** | 26.7504 | 2.9417 | 20.9847 – 32.5162 | **<0.001** | 25.4008 | 2.5415 | 20.4195 – 30.3820 | **<0.001** | 22.798 | 1.1235 | 20.5960 – 24.9999 | **<0.001** | 22.9378 | 1.5144 | 19.9695 – 25.9060 | **<0.001** | 23.0561 | 0.9491 | 21.1960 – 24.9162 | **<0.001** | 22.5554 | 1.0763 | 20.4459 – 24.6650 | **<0.001** | 20.4806 | 2.0995 | 16.3656 – 24.5955 | **<0.001** | 23.424 | 1.1446 | 21.1807 – 25.6673 | **<0.001** | 20.6469 | 1.3381 | 18.0242 – 23.2696 | **<0.001** |
| PREDICTOR | 0.0449 | 0.1226 | -0.1953 – 0.2851 | 0.714 | -3.1835 | 1.7881 | -6.6882 – 0.3211 | 0.075 | -0.4133 | 0.2902 | -0.9821 – 0.1556 | 0.154 | -0.0495 | 0.0454 | -0.1384 – 0.0394 | 0.275 | -0.1202 | 2.0547 | -4.1472 – 3.9069 | 0.953 | -0.0031 | 0.0128 | -0.0282 – 0.0220 | 0.807 | -1.4966 | 0.9515 | -3.3614 – 0.3683 | 0.116 | 0.5858 | 0.3153 | -0.0322 – 1.2038 | 0.063 | 0.4297 | 0.5778 | -0.7028 – 1.5622 | 0.457 | 0.1141 | 0.3307 | -0.5341 – 0.7622 | 0.73 | 0.8098 | 0.3894 | 0.0466 – 1.5729 | **0.038** |
| TIME | 0.9505 | 2.6651 | -4.2729 – 6.1739 | 0.721 | -0.1705 | 0.3671 | -0.8901 – 0.5491 | 0.642 | -0.651 | 0.844 | -2.3052 – 1.0031 | 0.44 | -1.245 | 0.7704 | -2.7549 – 0.2649 | 0.106 | 0.409 | 0.3322 | -0.2421 – 1.0601 | 0.218 | 0.8342 | 0.4429 | -0.0340 – 1.7023 | 0.06 | 0.4762 | 0.2822 | -0.0770 – 1.0294 | 0.092 | 0.8287 | 2.2064 | -3.4958 – 5.1532 | 0.707 | 2.8009 | 2.3387 | -1.7828 – 7.3846 | 0.231 | -1.8828 | 1.9787 | -5.7610 – 1.9953 | 0.341 | 3.7606 | 1.7916 | 0.2490 – 7.2721 | **0.036** |
| PREDICTOR * TIME | -0.0072 | 0.0349 | -0.0756 – 0.0612 | 0.836 | 1.1047 | 0.506 | 0.1130 – 2.0965 | **0.029** | 0.1091 | 0.0832 | -0.0540 – 0.2722 | 0.19 | 0.031 | 0.0136 | 0.0043 – 0.0576 | **0.023** | -0.0182 | 0.5792 | -1.1535 – 1.1171 | 0.975 | -0.0041 | 0.0034 | -0.0109 – 0.0026 | 0.228 | 0.1459 | 0.1307 | -0.1102 – 0.4021 | 0.264 | -0.6777 | 0.6041 | -1.8618 – 0.5064 | 0.262 | -0.0259 | 0.6546 | -1.3089 – 1.2572 | 0.968 | 0.7913 | 0.5534 | -0.2934 – 1.8760 | 0.153 | -0.7512 | 0.5378 | -1.8053 – 0.3029 | 0.16 |
| **Random effects** |  |  |  |  |  |  |  |  |  |  |  |  |  |  |  |  |  |  |  |  |  |  |  |  |  |  |  |  |  |  |  |  |  |  |  |  |  |  |  |  |  |  |  |  |
| σ2 (within-subject) | 322.76 | 17.966 | 309.398 - 336.9028 |  | 322.936 | 17.97 | 309.566 - 337.0932 |  | 322.846 | 17.968 | 309.478 - 336.9958 |  | 322.406 | 17.956 | 309.069 - 336.5223 |  | 322.76 | 17.966 | 309.396 - 336.9041 |  | 322.77 | 17.966 | 309.409 - 336.9122 |  | 322.612 | 17.961 | 309.253 - 336.7507 |  | 322.857 | 17.968 | 309.487 - 336.9957 |  | 322.928 | 17.97 | 309.549 - 336.0322 |  | 322.889 | 17.969 | 309.549 - 336.0322 | | 322.948 | 17.971 | 309.277 - 336.1483 |  |
| τ00:id (between-subject) | 30.6786 | 5.539 | 16.083 - 56.0977 |  | 26.5314 | 5.151 | 13.165 - 50.1554 |  | 28.9603 | 5.381 | 14.873 - 53.5834 |  | 30.5967 | 5.531 | 16.186 - 55.5326 |  | 30.9679 | 5.565 | 16.285 - 56.4897 |  | 30.9305 | 5.562 | 16.398 - 56.2491 |  | 28.9978 | 5.385 | 15.006 - 53.6301 |  | 30.8863 | 5.558 | 16.369 - 56.3814 |  | 29.4344 | 5.425 | 13.111 - 46.6177 |  | 30.3488 | 5.509 | 13.111 - 46.6177 | | 26.153 | 5.114 | 9.07 - 41.7691 |  |
| τ11: TIME (between-subject) | ####### | 1.453 | 0.892 - 4.3697 |  | ####### | 1.28 | 0.578 - 3.6596 |  | ####### | 1.381 | 0.759 - 4.0628 |  | ####### | 1.416 | 0.891 - 4.0522 |  | ####### | 1.449 | 0.872 - 4.3702 |  | ####### | 1.409 | 0.823 - 4.1559 |  | ####### | 1.42 | 0.834 - 4.235 |  | ####### | 1.423 | 0.857 - 4.2158 |  | ####### | 1.447 | 0.763 - 3.5229 |  | 1.937 | 1.392 | 0.763 - 3.5229 | | ####### | 1.417 | 0.583 - 3.1512 |  |
| **Model performance** |  |  |  |  |  |  |  |  |  |  |  |  |  |  |  |  |  |  |  |  |  |  |  |  |  |  |  |  |  |  |  |  |  |  |  |  |  |  |  |  |  |  |  |  |
| Deviance | 37680.2 |  |  |  | 37675.7 |  |  |  | 37678.1 |  |  |  | 37675 |  |  |  | 37680.3 |  |  |  | 37677.3 |  |  |  | 37677.8 |  |  |  | 37680 |  |  |  | 37679.2 |  |  |  | 37679.2 |  |  |  | 37677.4 |  |  |  |
| AIC | 37696.2 |  |  |  | 37691.7 |  |  |  | 37694.1 |  |  |  | 37691 |  |  |  | 37696.3 |  |  |  | 37693.3 |  |  |  | 37693.8 |  |  |  | 37696 |  |  |  | 37695.2 |  |  |  | 37695.2 |  |  |  | 37693.4 |  |  |  |
| log-Likelihood | -18840 |  |  |  | -18838 |  |  |  | -18839 |  |  |  | -18837 |  |  |  | -18840 |  |  |  | -18839 |  |  |  | -18839 |  |  |  | -18840 |  |  |  | -18840 |  |  |  | -18840 |  |  |  | -18839 |  |  |  |
